# *TaDA1*, a conserved negative regulator of kernel size, has an additive effect with *TaGW2* in common wheat (*Triticum aestivum* L.)

**DOI:** 10.1111/pbi.13298

**Published:** 2019-12-04

**Authors:** Hong Liu, Huifang Li, Chenyang Hao, Ke Wang, Yamei Wang, Lin Qin, Diaoguo An, Tian Li, Xueyong Zhang

**Affiliations:** ^1^ Key Laboratory of Crop Gene Resources and Germplasm Enhancement Ministry of Agriculture Institute of Crop Sciences Chinese Academy of Agricultural Sciences Beijing China; ^2^ Center for Agricultural Resources Research Institute of Genetics and Developmental Biology Chinese Academy of Sciences Shijiazhuang Hebei China; ^3^ Agronomy College Gansu Agriculture University Lanzhou, Gansu China

**Keywords:** *TaDA1*, kernel size, kernel weight, additive effect, *TaGW2*, *Triticum aestivum*

## Abstract

Kernel size is an important trait determining cereal yields. In this study, we cloned and characterized *TaDA1*, a conserved negative regulator of kernel size in wheat (*Triticum aestivum*). The overexpression of *TaDA1* decreased the size and weight of wheat kernels, while its down‐regulation using RNA interference (RNAi) had the opposite effect. Three *TaDA1‐A* haplotypes were identified in Chinese wheat core collections, and a haplotype association analysis showed that *TaDA1‐A‐HapI* was significantly correlated with the production of larger kernels and higher kernel weights in modern Chinese cultivars. The haplotype effect resulted from a difference in *TaDA1‐A* expression levels between genotypes, with *TaDA1‐A‐HapI* resulting in lower *TaDA1‐A* expression levels. This favourable haplotype was found having been positively selected during wheat breeding over the last century. Furthermore, we demonstrated that TaDA1‐A physically interacts with TaGW2‐B. The additive effects of *TaDA1‐A* and *TaGW2‐B* on kernel weight were confirmed not only by the phenotypic enhancement arising from the simultaneous down‐regulation of *TaDA1* and *TaGW2* expression, but also by the combinational haplotype effects estimated from multi‐environment field data from 348 wheat cultivars. A comparative proteome analysis of developing transgenic and wild‐type grains indicated that TaDA1 and TaGW2 are involved in partially overlapping but relatively independent protein regulatory networks. Thus, we have identified an important gene controlling kernel size in wheat and determined its interaction with other genes regulating kernel weight, which could have beneficial applications in wheat breeding.

## Introduction

Common wheat (*Triticum aestivum* L., AABBDD) is a major staple crop in the world. With growing global population and increasing demand for wheat, wheat yields must be further improved. Grain weight and size are major components of wheat yield and are therefore key targets for the further improvement of this crop (Li and Yang, 2017; Mohler *et al.*, 2016; Xie *et al.*, 2015). Many genes regulating wheat grain weights have been identified using comparative genomics approaches, including *TaGW2* (Qin *et al.*, 2017; Simmonds *et al.*, 2016; Su *et al.*, 2011), *Ta* *CKX2* (Zhang *et al.*, 2012), *TaGS5* (Ma *et al.*, 2016; Wang *et al.*, 2015), *TaCYP78A3* (Ma *et al.*, 2015), *TaTGW6* (Hanif *et al.*, 2016; Hu *et al.*, 2016), *TaFlo2‐A1* (Sajjad *et al.*, 2017), and *TaAGP‐S1* and *TaAGP‐L* (Hou *et al.*, 2017). Based on the knowledge of their counterparts in rice (*Oryza sativa*), these genes are predicted to function in diverse signalling pathways, including proteasomal degradation, phytohormone signalling, and starch biosynthesis (Zuo and Li, 2014). Although they are known to regulate grain size or grain filling, the interactions between these different regulators and the overall regulatory network underlying grain development remain to be elucidated.

The ubiquitin–proteasome pathway plays a key role in controlling organ size in dicot and monocot plants (Brinton *et al.*, 2018; Li and Li, 2016; Shi *et al.*, 2019). Recently, increasing evidence has indicated that the ubiquitin receptor DA1, a conserved component of the ubiquitin–proteasome pathway, has important functions in regulating seed size. In *Arabidopsis thaliana*, DA1 functions partially redundantly with one of its homologs, DA1‐RELATED 1 (DAR1), to control seed size by restricting the period of cell proliferation in maternal integuments (Dong *et al.*, 2017; Li *et al.*, 2008). The dominant‐negative mutant *da1‐1* carries a G‐to‐A mutation in *DA1*, causing an R‐to‐K change in the conserved amino acid at position 358 (DA1^R358K^). This *da1‐1* allele dramatically increases the seed and organ size of *Arabidopsis* plants because the mutant DA1^R358K^ protein negatively impacts the function of DA1 and DAR1 (Li *et al.*, 2008). In rapeseed (*Brassica napus*) and maize (*Zea mays*), the overexpression of a similar mutant form of *DA1* or *DAR1* homologs also improved their seed weights and organ sizes, thereby increasing the overall grain yield and biomass (Wang *et al.*, 2017a; Xie *et al.*, 2018). Furthermore, a loss‐of‐function T‐DNA *DA1* mutant (*da1‐ko1*) in *Arabidopsis* also caused an increase in seed size, indicating that *DA1* can be studied relatively independently of *DAR1* in regulating seed size (Dong *et al.*, 2017).

The RING finger‐type E3 ubiquitin ligases BIG BROTHER (BB) and DA2 restrict organ growth (Disch *et al.*, 2006; Xia *et al.*, 2013) and function synergistically with DA1 in organ size determination (Li *et al.*, 2008; Xia *et al.*, 2013). A recent study showed that BB and DA2 ubiquitylate DA1 and activate its peptidase activity, and then activated DA1 cleaves and destabilizes BB and DA2 in a feedback mechanism (Dong *et al.*, 2017). In addition, DA2 physically interacts with DA1, which may enable DA1 to recognize specific substrates for degradation (Xia *et al.*, 2013). DA2 shares significant sequence similarity with GW2, which is known to determine grain weight (Song *et al.*, 2007), and the overexpression of *DA2* and *OsGW2* in *Arabidopsis* both restricted seed growth, similar to the phenotype change in rice (Xia *et al.*, 2013). GW2 and its homologs have been confirmed to be conserved negative regulators of grain size and weight in various crops including rice (Song *et al.*, 2007), maize (Li *et al.*, 2010), and wheat (Qin *et al.*, 2017; Su *et al.*, 2011). Both DA1 and GW2 (or DA2) therefore have conserved functions in kernel size control which implies that their interaction in the ubiquitin–proteasome pathway may also be conserved in plants.

In this study, we isolated *DA1* in wheat using a bioinformatics approach and mapped the *TaDA1* homoeologs on chromosomes 2A, 2B, and 2D. Transgenic analysis showed that the overexpression of *TaDA1* in wheat causes a decline in kernel size and weight, while the down‐regulation of *TaDA1* using RNA interference (RNAi) had the opposite effect, indicating that *TaDA1* has a conserved function in the negative regulation of kernel size. Our haplotype association analysis also demonstrated that, in modern Chinese cultivars, the favourable haplotype of *TaDA1‐A* was significantly associated with higher kernel weights. Moreover, we found that TaDA1‐A physically interacted with TaGW2‐B and verified their additive effects not only through the enhanced kernel size phenotype generated by the simultaneous down‐regulation of *TaDA1* and *TaGW2* in wheat, but also using a combinational analysis of *TaDA1‐A* and *TaGW2‐B* haplotypes in the association population. Our findings provide evidence that *TaDA1* is an important gene controlling kernel size in wheat, and it can be potentially applied in combination with other yield‐related genes in wheat high‐yield breeding.

## Results

### Cloning and expression analysis of *TaDA1* homoeologs in wheat

Based on the reported *Arabidopsis* and maize *DA1* sequences (Li *et al.*, 2008; Xie *et al.*, 2018), we identified *DA1* homologs in wheat through a bioinformatics search on Ensembl Plants (IWGSC, 2018). *TaDA1* was identified as the closest homolog of *AtDA1* and *ZmDA1*, and its homoeologs were located on chromosomes 2A, 2B, and 2D (Table S1; Figure S1). The coding regions of *TaDA1‐A*, *‐B*, and *‐D* are 6363, 6660, and 6180 bp in length, respectively, and each consists of 11 exons and 10 introns (Figure S2). TaDA1‐A, ‐B, and ‐D all comprise 504 amino acids and share high sequence similarities with AtDA1 (~60%) and ZmDA1 (~86%), especially in the conserved UIM and LIM domains (Li *et al.*, 2008; Figure S3). This high level of evolutionary conservation suggests that *TaDA1* may have similar biological functions to *AtDA1* and *ZmDA1*.

**Figure 1:**
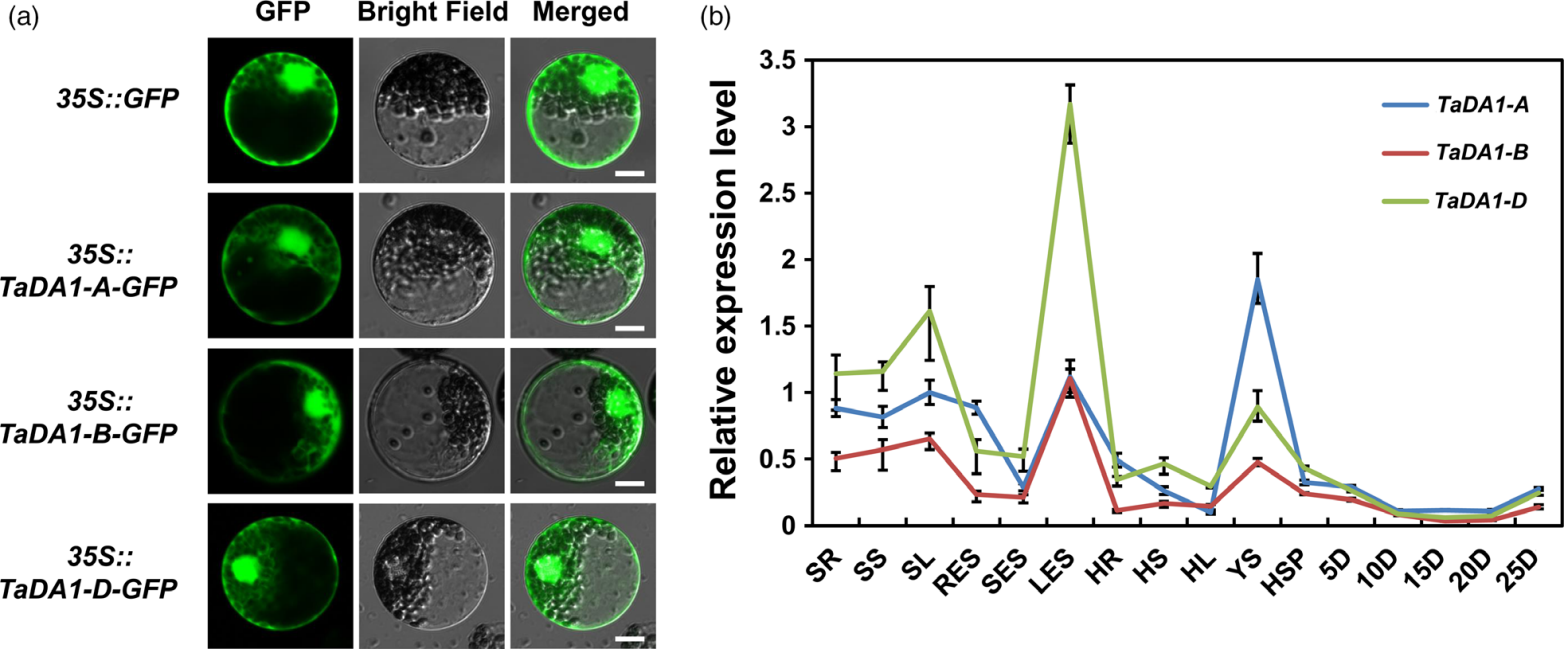
Characterization of *TaDA1* homoeologs. (a) Subcellular localization of TaDA1 homoeologs in wheat protoplasts. *GFP* and *TaDA1s‐GFP* fusions under the control of the CaMV 35S promoter were transiently expressed in wheat protoplasts. Bar = 10 µm. (b) Tissue expression patterns of the three homoeologs in various Chinese Spring tissues. SR, seedling roots; SS, seedling stems; SL, seedling leaves; RES, roots at elongation stage; SES, stems at elongation stage; LES, leaves at elongation stage; HR, roots at heading stage; HS, stems at heading stage; HL, leaves at heading stage; YS, 1–2‐cm young spikes; HSP, spikes at heading stage; 5D–25D, grains at 5–25 days post‐anthesis, respectively. Normalized values of *TaDA1* expression relative to *Actin* were given as mean ± SD from three replicates.

We first investigated the subcellular localization of TaDA1 in wheat protoplasts. The coding sequences (CDSs) of the *TaDA1* homoeologs were fused in‐frame with the gene encoding green fluorescent protein (GFP), and the resulting constructs were introduced into wheat protoplasts using the PEG method (Zheng *et al.*, 2014). As shown in Figure 1a, the GFP fluorescence patterns of the fused TaDA1‐A, ‐B, or ‐D proteins were distributed throughout the cytoplasm and nucleus, indicating that these TaDA1 homoeologs have similar subcellular localizations. Next, we analysed the tissue‐specific expression patterns of the *TaDA1* homoeologs using quantitative real‐time PCR (qRT‐PCR) with genome‐specific primers (Table S2). The *TaDA1* homoeologs were all constitutively expressed in various wheat tissues but showed higher expression levels in the leaves (seedling and elongation stages) and young spikes (Figure 1b). Among the three homoeologs, *TaDA1‐B* and *TaDA1‐D* were predominantly expressed in vegetative organs such as the leaves and roots, while *TaDA1‐A* was predominantly expressed in reproductive organs such as the young spikes, implying that they might play partially redundant but somewhat divergent roles in regulating the development of different organs.

### *TaDA1* negatively regulates kernel weight and size in wheat

**Figure 2:**
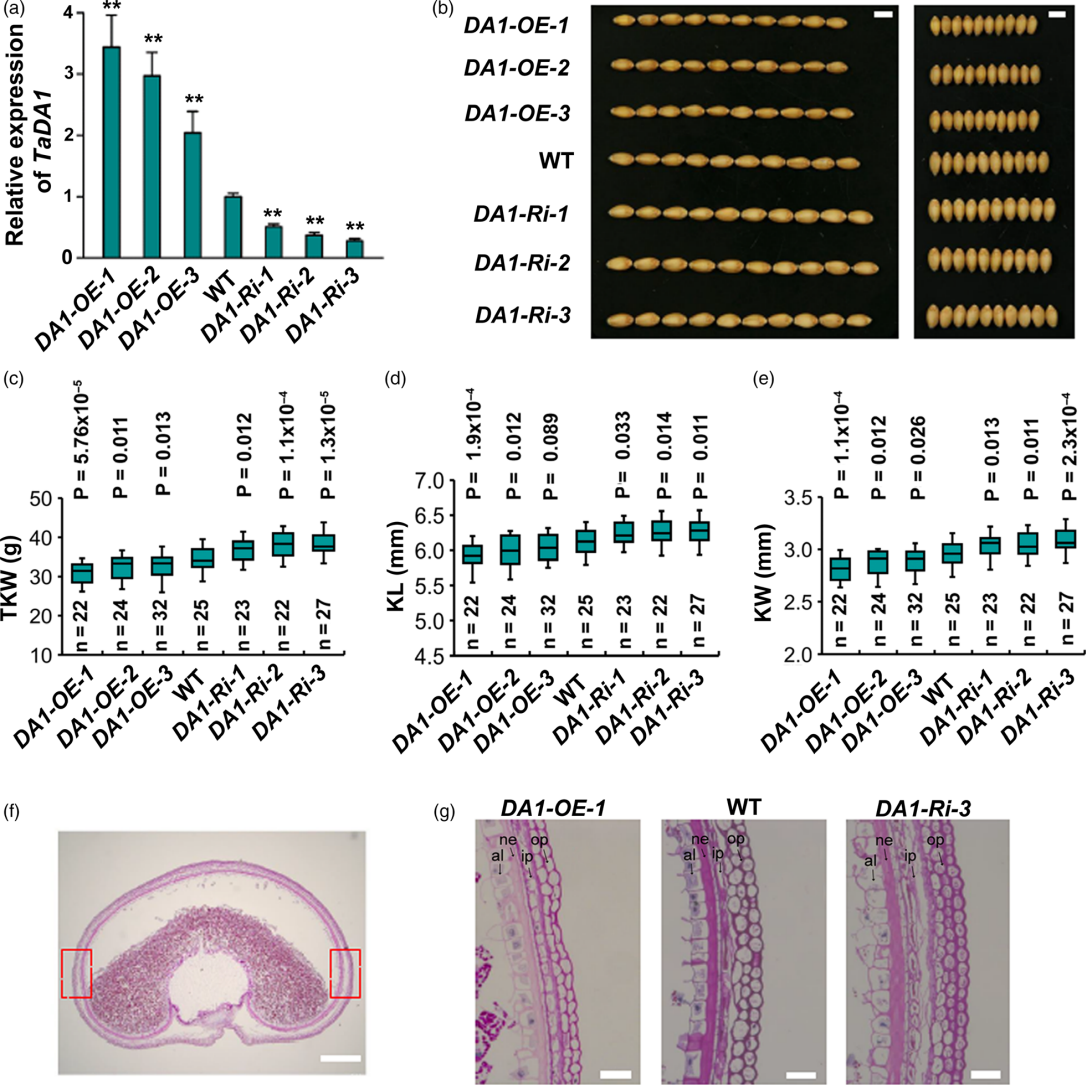
Phenotypic comparison of kernel traits among *TaDA1* overexpression lines, RNAi lines, and the WT under field conditions. (a) Expression levels of *TaDA1* in three representative T_2_ generation overexpression lines (*OE‐1*–*3*), RNAi lines (*Ri‐1*–*3*), and the WT. The values are presented as mean ± SD. **P < 0.01 (ANOVA) indicates a significant difference to the WT. (b) Comparison of the kernel phenotypes among these genotypes. Bar = 5 mm. (c) Thousand‐kernel weights (TKW), (d) kernel lengths (KL), and (e) kernel widths (KW) of these genotypes. Box plots showing the components of descriptive statistics including sample size (*n*), medians (black lines), upper and lower quartiles (box edges), minimums and maximums (whiskers), and *P* values (ANOVA). (f) A representative section of the middle portion of a developing grain of Fielder (WT) wheat at 15 DPA. Bar = 500 µm. (g) Comparison of the outer pericarp cells in the *TaDA1* overexpression line (*DA1‐OE‐1*), RNAi line (*DA1‐Ri‐3*), and WT. Each image was taken at the boxed areas indicated in (f). Op, outer pericarp; ip, inner pericarp; ne, nucellar epidermis; al, aleurone layer. Bars = 50 µm.

To study the physiological function of *TaDA1* in wheat, we generated *TaDA1‐A* overexpression lines and RNAi transgenic plants in the hexaploid wheat cultivar Fielder using *Agrobacterium*‐mediated transformation (Wang *et al.*, 2017b). The total expression levels of *TaDA1* were examined in the transgenic plants using qRT‐PCR. As shown in Figure 2a, the mRNA levels of *TaDA1* were significantly increased in all three representative overexpression lines (*OE‐1*–*3*; *P* < 0.01) and were reduced in the three independent RNAi lines (*Ri‐1*–*3*; *P* < 0.01). Compared with the WT control, the overexpression lines had smaller kernels, with 1.7–5.6% shorter kernel lengths (KLs) and 3.4–5.1% narrower kernel widths (KWs; Figure 2b–e). The RNAi lines produced significantly larger kernels than the WT, with approximately 2.5–3.5% longer KLs and 4.0–6.8% wider KWs when grown under field conditions (Figure 2b–e). Consistent with these results, the thousand‐kernel weight (TKW) of the overexpression lines and RNAi lines was 2.9–4.5 g lower and 3.6–5.9 g higher than that of the WT, respectively (Figure 2c). The phenotypic effects of the transgenic lines were negatively related to the expression levels of *TaDA1*; for example, the *OE‐1* line had the highest *TaDA1* expression level among the three overexpression lines and demonstrated the strongest reductions in kernel size and weight (Figure 2a–e). No significant differences were observed in other yield‐related traits between the transgenic lines and the WT, including the tiller number, grain number per spike, spike length, and spikelet number per spike (Figure S4). Our results show that the down‐regulation of *TaDA1* might be effective for improving wheat yields, and confirm that *TaDA1* is a conserved negative regulator of kernel size and weight.

### *TaDA1* restricts the proliferation of maternal pericarp cells

To test whether *TaDA1* affects maternal pericarp cell growth, we observed the outer layer cells in grains harvested at 15 days post‐anthesis (DPA) from *DA1*‐overexpressing plants (*DA1‐OE‐1*), *DA1* RNAi plants (*DA1‐Ri‐3*), and the WT (Figure 2f, g). Compared with the WT, the *DA1‐Ri‐3* plants produced more outer pericarp cells and consequently formed a wider pericarp cell layer (Figure 2g, Figure S5a). By contrast, the outer pericarps of the *DA1‐OE‐1* plants contained approximately 8% fewer cells than those of the WT (Figure 2g, Figure S5a). Furthermore, the cell length was slightly reduced in the outer pericarp of the *DA1‐Ri‐3* plants (Figure S5b). We also observed similar alteration of thickness in other cell layers including inner pericarp and nucellar epidermis among *DA1‐Ri‐3*, *DA1‐OE‐1,* and the WT (Figure 2g). These results confirmed that *TaDA1* has a conserved function in restricting maternal cell proliferation.

### *TaDA1‐A* haplotypes were associated with kernel weight and size in modern Chinese cultivars

**Figure 3:**
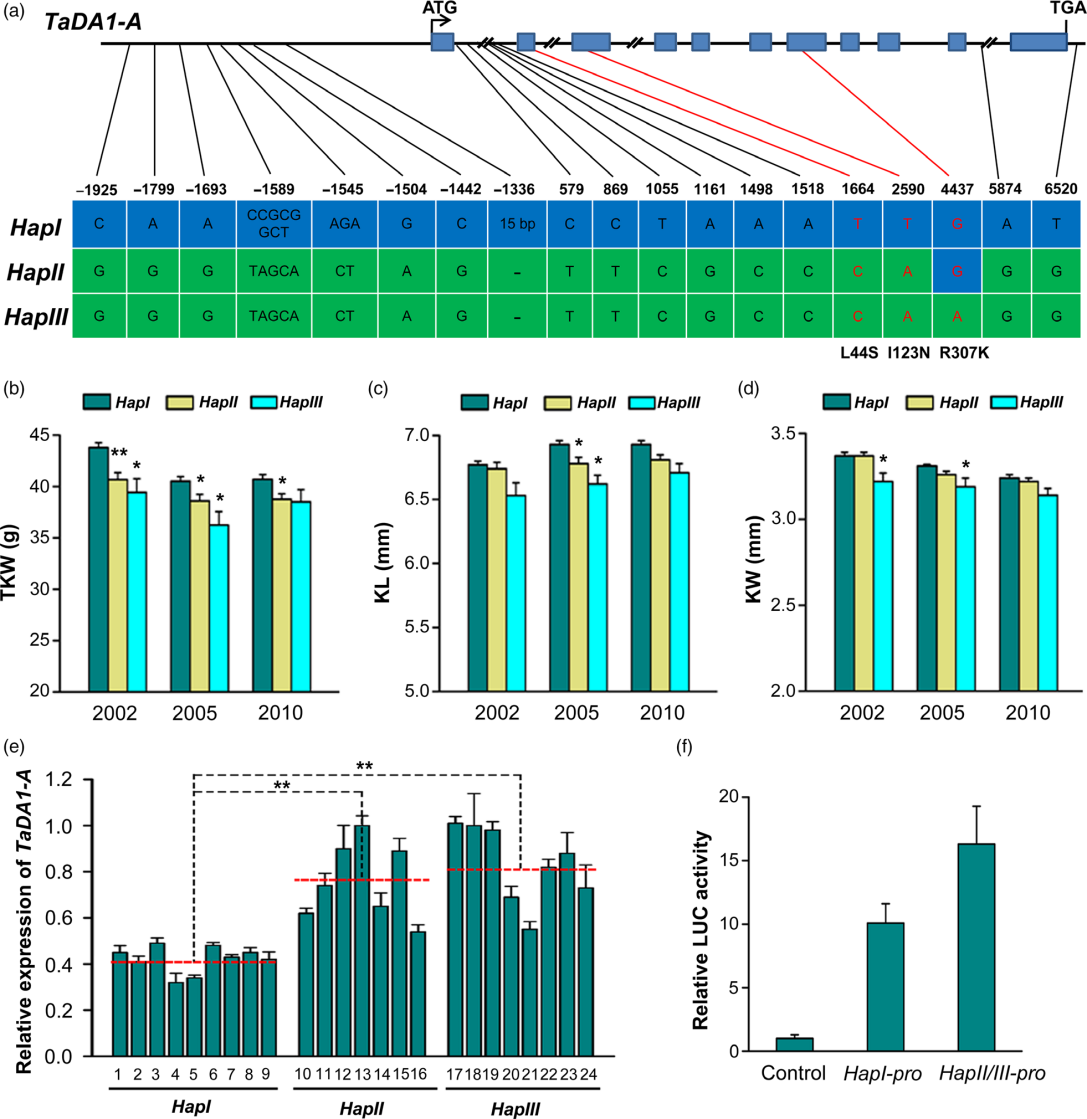
*TaDA1‐A* haplotype identification and haplotype association analysis with kernel traits in modern Chinese cultivars. (a) Variations in the *TaDA1‐A* promoter and genomic regions could be divided into three haplotypes (*TaDA1‐A‐HapI/II/III*). The blue boxes indicate exons. The SNPs in red cause an amino acid substitution (L44S, I123N, and R307K). (b–d) Association of *TaDA1‐A* haplotypes with the thousand‐kernel weights (TKW; b), kernel lengths (KL; c), and kernel widths (KW, d) of 348 modern Chinese cultivars in three environments (Luoyang, 2002; Luoyang, 2005; Shunyi, 2010). The number of *HapI*, *HapII,* and *HapIII* cultivars is 209, 116, and 23, respectively. The values are presented as mean ± SE. *P < 0.05 and **P < 0.01 (ANOVA) indicate significant differences from *HapI*. (e) Differential expression levels of *TaDA1‐A* in the 10‐DPA grains of 24 wheat accessions with different haplotypes. The red‐dashed lines indicate the average expression levels of each haplotype accession. 1, Shanxibaimai; 2, Baiqimai; 3, Xiaobaimang; 4, Dahongmai; 5, Huangguaxian; 6, Chushanbao; 7, Dabaimai; 8, Yuqiumai; 9, Chinese Spring; 10, Hongjinmai; 11, Xiaokouhong; 12, Daimanghongmai; 13, Zhuoludongmai; 14, Hongmai; 15, Youmangbaifu; 16, Shuilizhan; 17, Jiahongmai; 18, Lanhuamai; 19, Baiqiumai; 20, Chejianzi; 21, Baimangmai; 22, Banjiemang; 23, Bianbachunmai; and 24, Dianxihongkeyangmai. **P < 0.01 (ANOVA). (f) Relative LUC activity (LUC/REN) from the transient expression analysis of *TaDA1‐A* promoters with *HapI* and *HapII/III*. The blank pGreenII vector was used as control.

Since *TaDA1‐A* was the most highly expressed homoeolog in the reproductive organs, we concluded that it might play the largest role in affecting kernel weight and size. Specific primers were designed to obtain the genomic sequence of *TaDA1‐A*, including the 2.1‐kb promoter region and the 6.4‐kb coding region (Table S2). By sequencing 36 highly diverse Chinese wheat accessions (Table S3), we detected 19 natural variations present in the *TaDA1‐A* genomic regions, most of which were located in the introns or promoter regions (Figure 3a). Only three SNPs were located in exons, all of which caused amino acid substitutions (Figure 3a). These variations resulted in three distinct haplotypes (*TaDA1‐A‐HapI/II/III*) in the common wheat cultivars surveyed. Based on two of the exonic SNPs (T/A at 2,590 bp and G/A at 4,437 bp), we developed two dCAPS (derived cleaved amplified polymorphic sequence) markers to distinguish the three haplotypes of *TaDA1‐A* (Figure S6).

After using these dCAPS markers to genotype 348 modern Chinese cultivars released since the 1940s (Table S4), we performed an association analysis between the *TaDA1‐A* haplotypes and 11 agronomic traits collected in multiple environments. Consistent with its function in controlling kernel size and weight, the *TaDA1‐A* haplotypes were significantly correlated with TKW, KL, and KW (Figure 3b–d). The mean TKW of *TaDA1‐A‐HapI* was significantly higher than those of the *TaDA1‐A‐HapII/III* plants (3.1–4.3 g higher in 2002, 1.9–4.2 g higher in 2005, and 2.0–2.2 g higher in 2010; Figure 3b, Table S5). Similar results were found in KL and KW, except that no significant differences were observed in the KLs of *HapI* and *HapII* plants (Figure 3c, d). To further verify the results in the genetic populations, we detected an F_16_ segregating population derived from the cross Chuan 35050 × Shannong 483 (Xu *et al.*, 2017). The mean TKW of *TaDA1‐A‐HapI* over four environments was significantly higher than that of *TaDA1‐A‐HapII* (Figure S7). In summary, these results indicate that *TaDA1‐A‐HapI* is the favourable haplotype because it is associated with larger kernels and higher TKWs in modern Chinese cultivars.

### Variations in the promoter region of *TaDA1‐A* lead to significant differences in expression among haplotypes

To explore the possible reasons for these distinct haplotype effects, we first focused on the three exonic SNPs, all of which caused amino acid changes (L44S, I123N, and R307K, respectively; Figure 3a, Figure S8a). Because the L44S and I123N mutations were near the UIM domains responsible for the ubiquitin‐binding activity of DA1 (Li *et al.*, 2008), we expressed maltose‐binding protein (MBP)‐fused *N*‐terminal TaDA1‐A‐HapI/II/III proteins (amino acids 1–140) containing the UIM domains in *Escherichia coli*. After incubation with ubiquitin‐agarose beads, no significant differences in the ubiquitin‐binding activity were detected among the HapI/II/III proteins, indicating that the L44S and I123N mutations did not affect TaDA1‐A ubiquitin binding (Figure S8b). We further expressed the full‐length HapI/II/III proteins in *Schizosaccharomyces pombe* using the pREP1 vector, as described previously (Lin *et al.*, 2012; Maundrell, 1990). The HapI/II/III proteins were all similarly able to inhibit cell division in these assays (Figure S8c); therefore, our results reduce the possibilities that the exonic SNPs are responsible for the phenotypic differences among the *TaDA1‐A* haplotypes.

As reported previously (Hou *et al.*, 2017; Su *et al.*, 2011), many of the natural variations present in promoter regions affect gene expression. It is therefore also possible that large variations in the *TaDA1‐A* promoter region cause the observed haplotype effects by modifying the expression level of this gene. To test this hypothesis, we examined *TaDA1‐A* expression in the 10‐DPA grains of wheat accessions with different *TaDA1‐A* haplotypes. As shown in Figure 3e, *TaDA1‐A* expression was significantly lower in the *HapI* accessions than in the *HapII* and *HapIII* accessions, consistent with the observation that *TaDA1* is a negative regulator of kernel size and *HapI* is associated with a higher TKW and larger kernels. We further compared *HapI* and *HapII*/*III* promoter driven luciferase (LUC) activities through transient expression assay in tobacco (*N. benthamiana*) leaves as described (Hellens *et al.*, 2005). As expected, the *HapI* promoter had significantly lower relative LUC activity (LUC/REN) compared with the *HapII/III* promoter (Figure 3f). These results suggest that variations in the *TaDA1‐A* promoter region cause the main haplotype effects in modern Chinese cultivars.

### *TaDA1‐A‐HapI* underwent positive selection during modern wheat breeding

The geographic distribution of the *TaDA1‐A* haplotypes was evaluated in both landraces and modern cultivars from China’s ten ecological zones (Hou *et al.*, 2014; Ma *et al.*, 2016). The frequency of the favourable haplotype *HapI* was increased in the modern cultivars relative to the landraces in the major Chinese production zones (Figure S9a, b). By contrast, the frequency of *HapIII* declined during the transition from landraces to modern cultivars. We next investigated the frequency change of the *TaDA1‐A* haplotypes during Chinese wheat breeding since the 1940s. The frequency of *HapI* showed a dramatic increase in the 1950s and has remained almost constant (~60%) since the 1960s, whereas the frequency of *HapIII* continuously decreased over the same period and almost disappeared after the 1980s (Figure S9c). In addition, *HapI* has become the dominant haplotype in the modern cultivars around the world, especially those developed in Europe or by CIMMYT (the International Maize and Wheat Improvement Center; Figure S9d). The presence of the favourable *TaDA1‐A‐HapI* haplotype is consistent with the yields of these lines (FAO Data, http://www.fao.org/docrep/006/y4011e/y4011e04.htm#TopOfPage). These results provide strong evidence that *TaDA1‐A‐HapI* underwent positive selection in global wheat breeding for higher yields.

### TaDA1‐A physically interacts with TaGW2‐B

**Figure 4:**
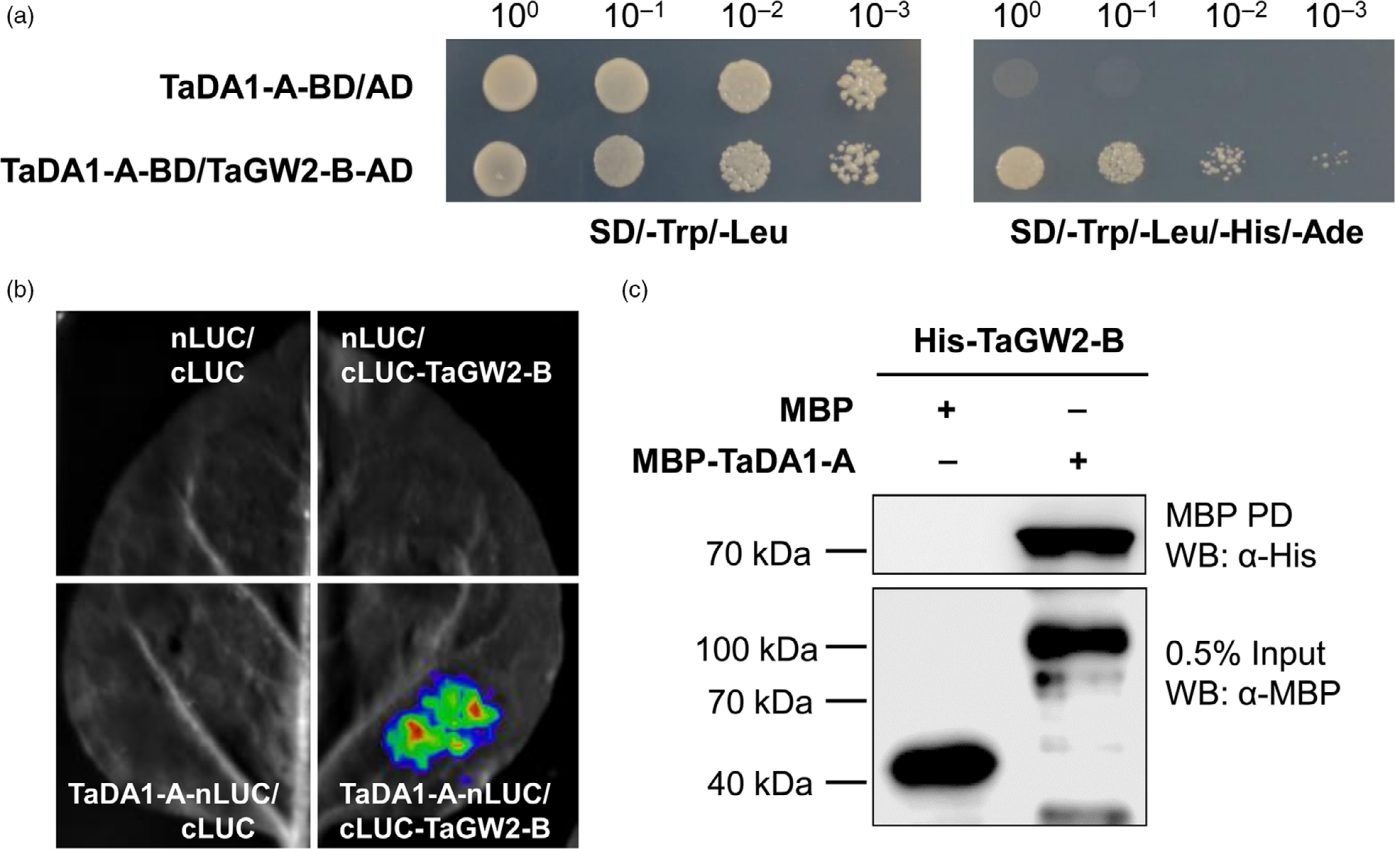
Protein–protein interactions between TaDA1‐A and TaGW2‐B. (a) Yeast two‐hybrid assay showing the interaction between TaDA1‐A and TaGW2‐B. BD, GAL4 DNA binding domain; AD, GAL4 activation domain. The yeast strains were serially diluted (10^0^–10^‐3^) before spotting on selection medium. SD/‐Trp/‐Leu, synthetic dextrose medium lacking Trp and Leu; SD/‐Trp/‐Leu/‐His/‐Ade, synthetic dextrose medium lacking Trp, Leu, His and Ade. (b) LCI assay showing that TaDA1‐A physically interacts with TaGW2‐B in tobacco cells. nLUC, *N*‐terminal part of LUC; cLUC, C‐terminal part of LUC. The nLUC and cLUC derivative constructs were transformed into *A. tumefaciens* and then co‐infiltrated into tobacco (*N. benthamiana*). The LUC signals were collected 48–72 h after infiltration. (c) *In vitro* pull‐down assay showing that MBP‐TaDA1‐A, but not MBP itself, can pull down the His‐TaGW2‐B protein.

In *Arabidopsis*, DA1 physically interacts with DA2, a RING finger‐type E3 ubiquitin ligase (Xia *et al.*, 2013). Because DA2 shares significant sequence similarity with GW2 in rice and wheat (Figure S10), we inferred that TaDA1 might also interact with TaGW2 in wheat. Using a yeast two‐hybrid system, we confirmed that TaDA1‐A could interact with TaGW2‐B (Figure 4a). To further verify that TaDA1‐A interacts with TaGW2‐B, we performed a firefly luciferase complementation imaging (LCI) assay in tobacco (*N. benthamiana*) cells. The co‐infiltration of TaDA1‐A‐nLUC and cLUC‐TaGW2‐B resulted in luminescence generated by the complemented luciferase, providing further evidence that TaDA1 and TaGW2 interact *in vivo* (Figure 4b). We also performed an *in vitro* pull‐down assay, which revealed that MBP‐TaDA1‐A but not the MBP control interacted with His‐TaGW2‐B (Figure 4c). These results confirmed that TaDA1‐A physically interacts with TaGW2‐B.

### The simultaneous silencing of *TaDA1* and *TaGW2* has an additive effect on kernel size and weight

In wheat, there is increasing evidence to suggest that *TaGW2* is a dominant‐negative regulator of kernel size and weight (Hong *et al.*, 2014; Qin *et al.*, 2017; Su *et al.*, 2011; Zhang *et al.*, 2018a). To confirm this effect, we generated *TaGW2* RNAi lines in the hexaploid wheat cultivar Fielder, which produced much larger kernels than the WT (5.7–7.8% larger in KL and 16.1–20.8% larger in KW; Figure S11a–e). Accordingly, the TKW of the *TaGW2* RNAi lines was 9.5–12.7 g higher than the WT (Figure S11c), showing that *TaGW2* silencing had a stronger effect than *TaDA1* silencing on increasing TKW (Figure 2c).

**Figure 5:**
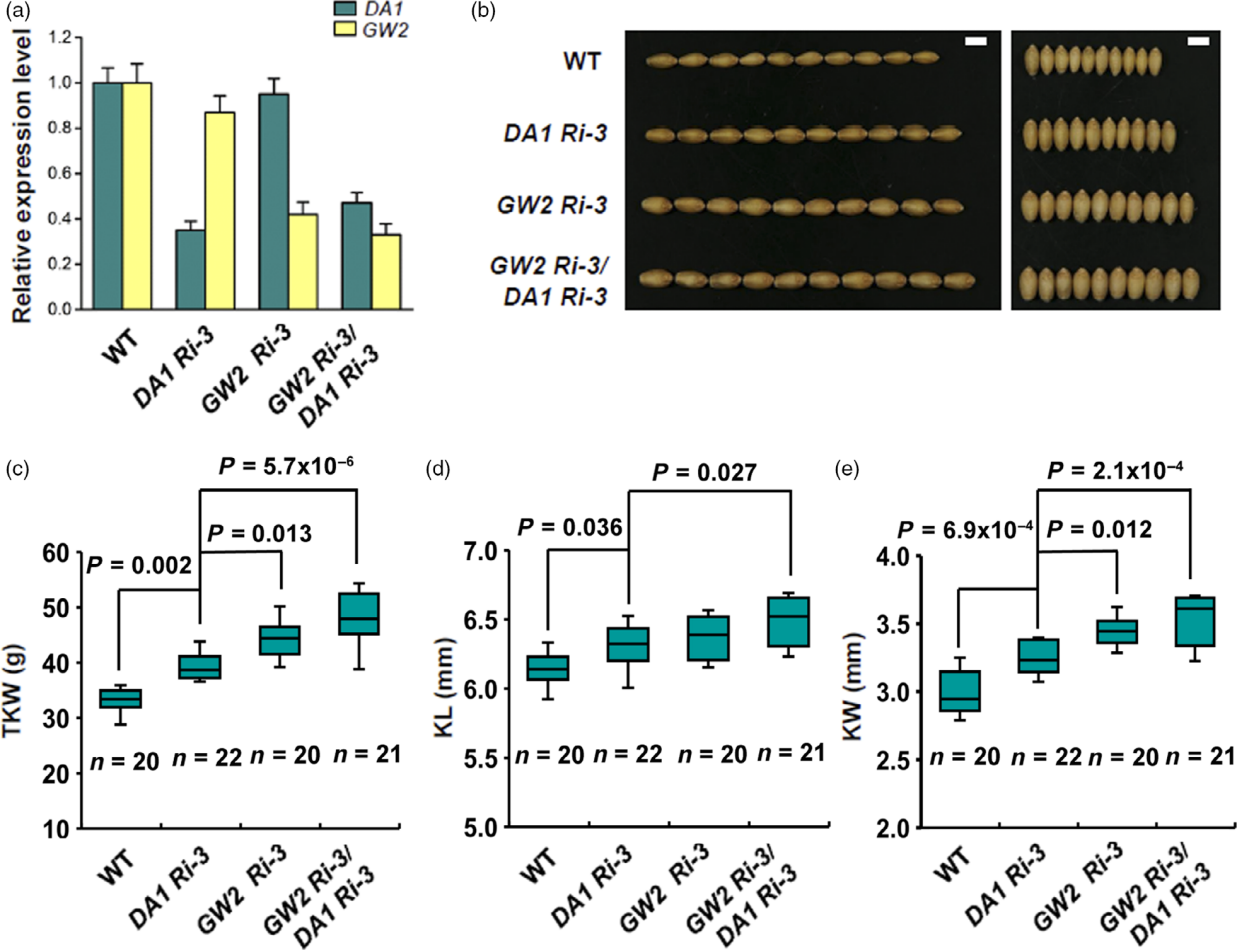
Genetically additive effect of *TaDA1* and *TaGW2* on kernel size and weight in wheat. (a) Expression levels of *TaDA1* and *TaGW2* in WT, the *TaDA1* RNAi line (*DA1‐Ri‐3*), *TaGW2* RNAi line (*GW2‐Ri‐3*), and double RNAi line (*GW2‐Ri‐3*/*DA1‐Ri‐3*). (b) Comparison of kernel phenotypes between these genotypes. Bar = 5 mm. (c) Thousand‐kernel weights (TKW), (d) kernel lengths (KL), and (e) kernel widths (KW) of these genotypes. Box plots showing the sample size (*n*), medians (black lines), upper and lower quartiles (box edges), minimums and maximums (whiskers), and *P* values (ANOVA).

To study the genetic interactions of *TaDA1* and *TaGW2*, we simultaneously silenced *TaDA1* and *TaGW2* in wheat by crossing the *GW2‐Ri‐3* and *DA1‐Ri‐3* transgenic lines (Figure 5a). The phenotypic results showed that both the *GW2‐Ri‐3*/*DA1‐Ri‐3* and *GW2‐Ri‐3* plants had larger kernels and higher TKWs than the *DA1‐Ri‐3* plants, but *GW2‐Ri‐3*/*DA1‐Ri‐3* showed a more significant increase in TKW and KW (Figure 5b–e). Compared with *DA1‐Ri‐3*, *GW2‐Ri‐3*/*DA1‐Ri‐3* showed a 20% increase in TKW and an 11% increase in KW (Figure 5c–e). Therefore, *GW2‐Ri‐3*/*DA1‐Ri‐3* had a greater increase in kernel size and TKW than both *DA1‐Ri‐3* and *GW2‐Ri‐3*, suggesting that *TaDA1* and *TaGW2* have genetically additive effects on these traits.

### The *TaDA1‐A* and *TaGW2‐B* haplotypes have additive effects on TKW

**Figure 6:**
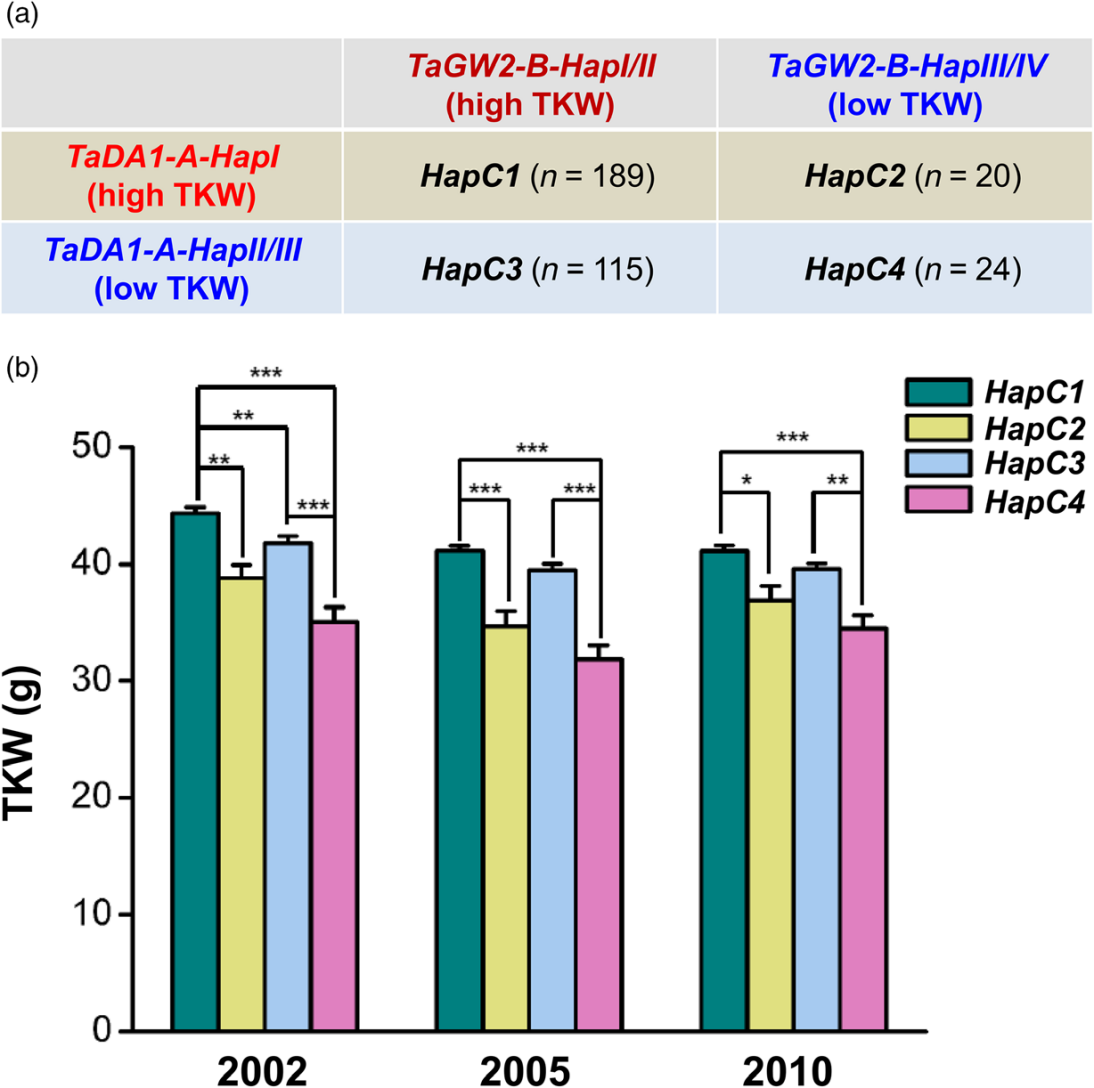
Combination of the *TaDA1‐A* and *TaGW2‐B* haplotypes with different effects on thousand‐kernel weights (TKW) in wheat. (a) Combinations of *TaDA1‐A* and *TaGW2‐B* high‐TKW and low‐TKW haplotypes (*HapC1–C4*). The number of *HapC1–C4* is 189, 20, 115, and 24, respectively, in 348 modern Chinese cultivars. (b) Association of combinational haplotypes (*HapC1–C4*) with TKW in 348 modern Chinese cultivars grown in three environments (Luoyang, 2002; Luoyang, 2005; Shunyi, 2010). The values are presented as mean ± SE. *P < 0.05, **P < 0.01, and ***P < 0.001 (ANOVA) indicate significant differences between these combinations.

Because both TaDA1 and TaGW2 are negative regulators of kernel size and weight and can physically interact with each other, we speculated that the *TaDA1‐A* and *TaGW2‐B* haplotypes may have a combinational effect on TKW. The results of our present and previous association studies (Qin *et al.*, 2017) revealed that the *TaDA1‐A* and *TaGW2‐B* haplotypes can both be divided into high‐TKW haplotypes (*TaDA1‐A‐HapI* and *TaGW2‐B‐HapI/II*) and low‐TKW haplotypes (*TaDA1‐A‐HapII/III* and *TaGW2‐B‐HapIII/IV*). Wheat accessions displaying these four combinations were divided into groups of high‐/high‐ (*TaDA1‐A/TaGW2‐B*), high‐/low‐, low‐/high‐, and low‐/low‐TKW haplotypes, designated *HapC1*–*C4,* respectively (Figure 6a, Table S4). Among the four combinations, *HapC1* (high‐*TaDA1‐A*/high‐*TaGW2‐B*) was associated with the highest TKW in the modern Chinese cultivars (44.4 g, 41.1 g, and 41.1 g in 2002, 2005, and 2010, respectively), while the TKW of *HapC4* (low‐*TaDA1‐A*/low‐*TaGW2‐B*) was the lowest among four haplotypes (Figure 6b), demonstrating an additive effect for the low‐ and high‐*TaDA1‐A* and *TaGW2‐B* haplotypes. Although the difference was not significant, the TKW of *HapC3* (low‐*TaDA1‐A*/high‐*TaGW2‐B*) was higher than that of *HapC2* (high‐*TaDA1‐A*/low‐*TaGW2‐B*), implying that the *TaGW2‐B* haplotypes had a stronger effect on TKW than the *TaDA1‐A* haplotypes (Figure 6b). Our data demonstrate the presence of an additive effect of the *TaDA1‐A* and *TaGW2‐B* haplotypes on the regulation of TKW.

### TaDA1 and TaGW2 function in partially overlapping but relatively independent protein networks

**Figure 7:**
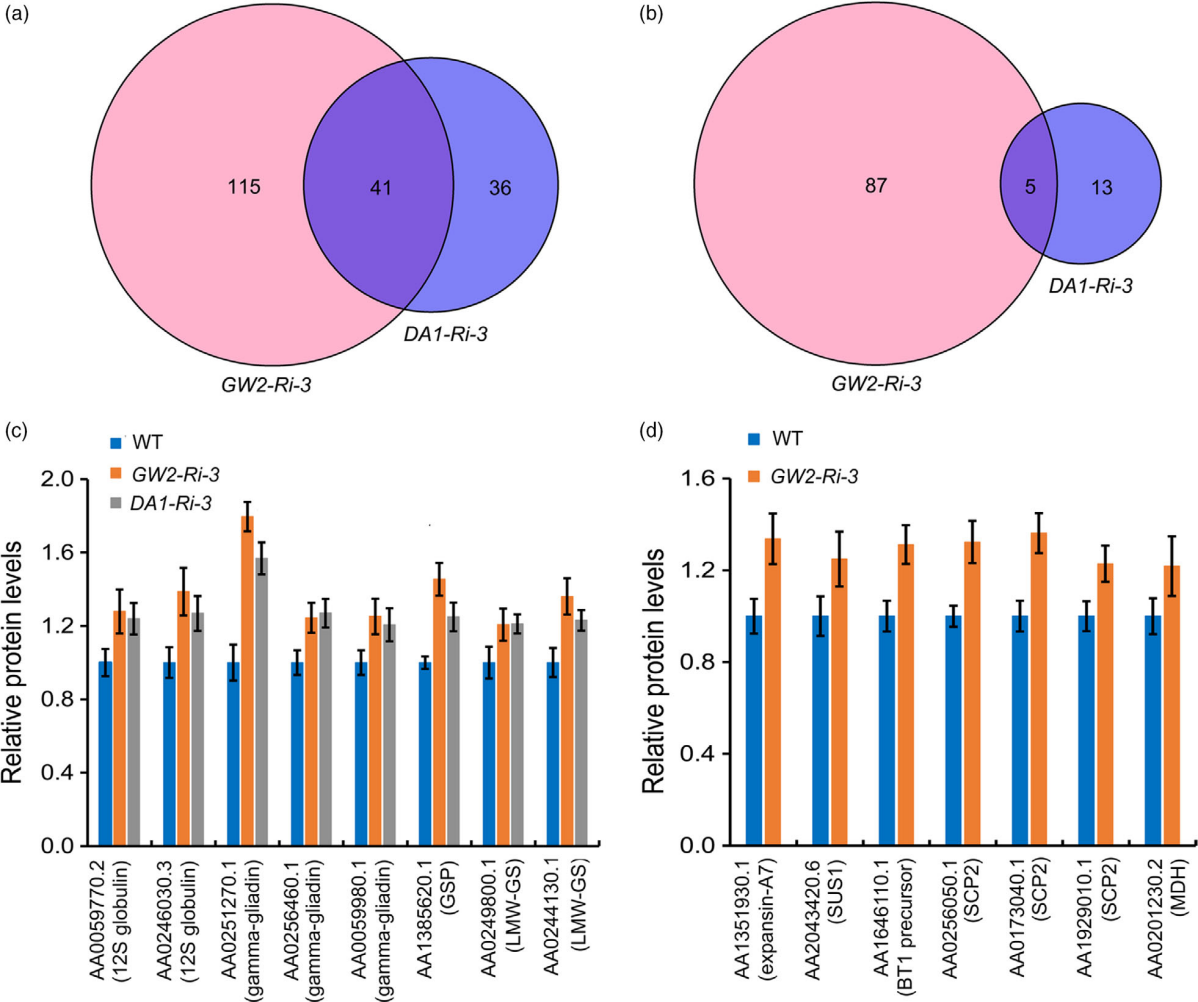
Comparative proteome analysis identified differently abundant proteins in 10‐DPA grains of *DA1‐Ri‐3* and *GW2‐Ri‐3*. (a, b) Number of up‐regulated (a) and down‐regulated (b) proteins in *DA1‐Ri‐3* and *GW2‐Ri‐3* compared with the WT control. (c) Representative up‐regulated proteins identified both in *DA1‐Ri‐3* and *GW2‐Ri‐3*. 12S globulin, 12S seed storage globulin 1; GSP, grain softness protein; LMW‐GS, low‐molecular‐weight glutenin subunit. (d) Representative up‐regulated proteins identified only in *GW2‐Ri‐3*. SUS1, sucrose synthase 1; BT1, ADP‐glucose brittle‐1 transporter precursor; SCP2, serine carboxypeptidase 2; MDH, malate dehydrogenase. The values are presented as mean ± SD.

TaDA1 and TaGW2 are both involved in the ubiquitin–proteasome pathway; therefore, their functions are related to substrate degradation. We performed a comparative proteome assay to identify proteins affected by TaDA1 and TaGW2. In grains at 10 DPA, a total of 95 differently abundant proteins were identified between WT and *DA1‐Ri‐3*, 77 of which were up‐regulated and 18 down‐regulated in *DA1‐Ri‐3* (Figure 7a, b; Table S6). By contrast, 249 differently abundant proteins were identified between the WT and *GW2‐Ri‐3*, including 157 up‐regulated proteins and 92 down‐regulated proteins in *GW2‐Ri‐3*. This indicated that *TaGW2* silencing evoked a more significant alteration of the abundance of downstream proteins than *TaDA1* silencing, which correlated with the finding that *TaGW2* had a more dominant effect on TKW than *TaDA1*.

*DA1‐Ri‐3* and *GW2‐Ri‐3* were found to share 41 up‐regulated proteins and five down‐regulated proteins relative to the WT (Figure 7a, b), which indicated that TaDA1 and TaGW2 function in partially overlapping regulatory pathways. The up‐regulated proteins were mainly involved in the biosynthesis of seed storage proteins, including multiple 12S seed storage globulins, low‐molecular‐weight glutenin subunits, gamma‐gliadins, and grain softness protein (Figure 7c, Table S6). Moreover, many of the differently abundant proteins were unique to *DA1‐Ri‐3* or *GW2‐Ri‐3*, indicating that TaDA1 and TaGW2 act relatively independently of each other; for example, several proteins related to cell proliferation and cell expansion were significantly up‐regulated in *GW2‐Ri‐3* (Figure 7d, Table S6). Three serine carboxypeptidases were up‐regulated in *GW2‐Ri‐3.* This group of proteins was previously reported to promote cell division and act as positive regulators of kernel size (Ma *et al.*, 2016). Expansin A7 was also found to be up‐regulated in *GW2‐Ri‐3* (Figure 7d). The expansin proteins are highly related to cell expansion, and expansin‐like A1 was the first verified substrate of GW2 in rice (Choi *et al.*, 2018). Furthermore, several crucial proteins involved in starch synthesis are also up‐regulated in *GW2‐Ri‐3*, including ADP‐glucose brittle‐1 transporter precursor and sucrose synthase 1 (Figure 7d).

## Discussion

In plants, seed size is a critical trait controlling seedling vigour and early growth, but it is also a key factor in determining grain yields in crops. Recently, the ubiquitin–proteasome pathway was identified as a conserved regulatory pathway of seed size in model plants (Li and Li, 2016). DA1, a core component of this regulatory pathway, acts partially redundantly with DAR1 to control seed size (Dong *et al.*, 2017; Li *et al.*, 2008). In this study, we cloned the *DA1* homoeologs in wheat and revealed their high protein sequence similarities to AtDA1, especially in the functional domains such as the UIMs and LIM (Figure S3), implying that they have similar protein structures and functions. Disruption of the *DA1* genes causes similar phenotypes in *Arabidopsis* and wheat, demonstrating their functional similarity; *da1‐ko1*, a loss‐of‐function T‐DNA mutant in *Arabidopsis*, produced larger seeds than the WT (Dong *et al.*, 2017), while silencing of *TaDA1* using RNAi also led to larger kernels in transgenic wheat (Figure 2b). AtDA1 was previously shown to physically interact with AtDA2 (Xia *et al.*, 2013), while TaDA1 was shown to interact with TaGW2 (Figure 4a–c). AtDA2 and TaGW2 are similar RING‐type E3 ubiquitin ligases (Figure S10); therefore, TaDA1 interacts with similar proteins to AtDA1. These findings suggest that DA1 is a conserved negative regulator of seed size in dicots and monocots, with a high level of conservation that facilitates the elucidation of its role and its application in crop yield research. Many other components of the ubiquitin–proteasome pathway share conserved sequences and functions (Li and Li, 2016); therefore, future research should build on these characterized proteins to elucidate the function of other conserved proteins in the ubiquitin–proteasome pathway.

Many variations identified in mutant studies result in dramatic changes of protein functions, including missense mutations, premature stop codon mutations, and frame‐shift mutations. Unlike artificial mutations, most natural variations with a phenotypic effect are located in regulatory regions such as the promoters and introns. Previous studies of *DA1* focused on the AtDA1^R358K^ mutation in the *da1‐1* dominant‐negative mutant. This mutation inhibited the activities of DA1 and DAR1, significantly increasing seed sizes in *Arabidopsis*, as well as transgenic rapeseed and maize (Li *et al.*, 2008; Wang *et al.*, 2017a; Xie *et al.*, 2018); however, this mutation has not been identified in natural variations so far. In this study, we sequenced many natural variations in *TaDA1‐A*, most of which were located in the promoter and intronic regions (Figure 3a). Although we identified three exonic SNPs that introduced missense mutations, they did not cause obvious changes in the functions of the mutant TaDA1‐A proteins (Figure S8). By contrast, the variations in the promoter regions altered the expression level of *TaDA1‐A*, providing a convincing explanation for the phenotypic differences among the three *TaDA1‐A* haplotypes (Figure 3a, b). Similar regulatory patterns have also been observed in many other yield‐related genes, including *TaGW2‐A* (Su *et al.*, 2011), *TaGW2‐B* (Qin *et al.*, 2017), and *TaAGP‐L‐B* (Hou *et al.*, 2017). Variations in regulatory regions are increasingly believed to be advantageous for the rapid adaptation of crops by enabling gene expression levels to be fine‐tuned to their environments; therefore, these variations are potential target sites in future wheat breeding and selection.

Marker‐assisted selection (MAS) is a highly efficient method for the genetic improvement of crops. The process of wheat breeding largely involves the accumulation of favourable alleles or haplotypes and can therefore be dramatically sped up using MAS (Gupta *et al.*, 2010). In this study, we developed two dCAPS markers to distinguish three haplotypes of *TaDA1‐A* (Figure S6). Our association analysis indicated that the *TaDA1‐A* haplotypes were significantly correlated with differences in the kernel size and weights of modern Chinese cultivars (Figure 3b–d). *TaDA1‐A‐HapI*, the favourable haplotype producing larger kernel sizes and higher TKWs, was gradually accumulated over the course of the wheat breeding process (Figure S9a–c); however, further increases in the frequency of this haplotype are still possible as it is not yet saturated in modern cultivars (Figure S9c). Our *TaDA1‐A* molecular markers therefore have the potential to be applied in the MAS breeding for high TKWs in wheat. Previous studies have confirmed that combinations of favourable alleles or haplotypes associated with higher TKWs show strong addictive effects, including *TaGW2‐A‐HapA* and *TaGW2‐B‐HapI* (Qin *et al.*, 2017), *TaSus2‐A‐HapA* and *TaSus1‐B‐HapT* (Hou *et al.*, 2014), and *TaAGP‐S1‐A‐HapI* and *TaAGP‐L‐B‐HapI* (Hou *et al.*, 2017). TaDA1‐A physically interacts with TaGW2‐B, providing a strong basis for the study of their genetically additive effects. We revealed that the combinational effect of the favourable *TaDA1‐A* and *TaGW2‐B* haplotypes (*TaDA1‐A‐HapI* and *TaGW2‐B‐HapI/II*) was significantly higher than the phenotypic effect of any single favourable haplotype (Figure 6a, b). In conclusion, pyramiding favourable haplotypes of *TaDA1‐A*, *TaGW2‐B*, and other major genes controlling yield‐related traits, will be more effective in future wheat yield improvement.

*TaGW2* showed a stronger effect than *TaDA1* on kernel size and weight in wheat, both in the haplotype association analysis and in a transgenic phenotypic assay (Figure 5b–e). The likely explanation is that DA1 acts partially redundantly with its homologs such as DAR1 (Li *et al.*, 2008), and that the down‐regulation of *DA1* alone has only a limited impact on the activities of its related proteins. Despite this, manipulating *TaDA1* expression still alters kernel traits in wheat (Figure 2a–e), indicating that *DA1* and its homologs are potential target genes for the future breeding of high‐yielding wheat cultivars. Recently, gene editing has been widely applied to improve the agronomic traits of crops (Wang *et al.*, 2014; Zhang *et al.*, 2018a). It is possible that this technique can be used to knock out *DA1* and its close homologs (Zhang *et al.*, 2018b), potentially generating a much greater impact on wheat kernel size. Furthermore, our proteomic data showed that TaDA1 and TaGW2 affected partially overlapping but relatively independent protein networks involved in diverse pathways (Figure 7a, b), including protein and starch biosynthesis, cell proliferation, and cell expansion. These proteins might promote rapid grain filling and enhance the final kernel size and TKW of the transgenic plants. Further work will be needed to identify the specific substrates of DA1 and GW2 and clarify their functional mechanisms in regulating kernel size in wheat.

## Experimental procedures

### Plant materials and phenotype assessment

A total of 505 Chinese wheat accessions including 157 landraces and 348 modern cultivars were used for the marker screening and association analyses, as previously described (Hou *et al.*, 2017). Agronomic trait data for these wheat accessions were collected from plants grown in Luoyang, Henan Province, China in 2002 and 2005, and in Shunyi, Beijing, China in 2010 (Table S4). The field planting design and agronomic trait evaluation were described previously (Su *et al.*, 2011). In addition, 384 European, 429 North American, 53 CIMMYT (the International Maize and Wheat Improvement Center), 82 former USSR, and 51 Australian modern wheat cultivars were used to investigate the global distribution of *TaDA1‐A* haplotypes in cultivars released during the last century (Hou *et al.*, 2014). For tissue‐specific expression analysis, different tissue samples were collected from wheat plants grown in the field at seedling stage, elongation stage, and heading stage, respectively. The *TaDA1* and *TaGW2* transgenic wheat and wild type were grown in the field at the CAAS experimental station in Beijing under natural conditions. For each T_2_ generation transgenic line, the phenotypes of at least 20 plants were analysed. Kernel traits of transgenic lines were measured after harvesting according to Su *et al. *(2011).

### RNA extraction and qRT‐PCR analysis

Total RNA was extracted from various Chinese Spring tissues using an RNAprep Pure Plant Kit (Tiangen Biotech, Beijing, China). The cDNA was synthesized using the FastQuant RT Kit (Tiangen Biotech) and subjected to qRT‐PCR analysis on a LightCycler 96 Real‐Time PCR system (Roche Applied Science, Penzberg, Germany) using the SYBR Premix Ex Taq (Takara Bio, Beijing, China), as previously described (Ma *et al.*, 2016). Genome‐specific primers of *TaDA1* homoeologs were validated by sequencing. The wheat *Actin* gene was used as the internal reference (Li *et al.*, 2015). The relative expression of each gene was calculated as a fold change using the comparative *C*_T_ method (Livak and Schmittgen, 2001). All assays were performed three times in independent experiments. Primers used in this study are listed in Table S2.

### Subcellular localization

The full‐length CDSs of the *TaDA1* homoeologs were amplified from Chinese Spring seedling cDNA sample and sub‐cloned into the pJIT163‐GFP vector (Zheng *et al.*, 2014). The verified fusion constructs and the control pJIT163‐GFP vector were introduced into wheat mesophyll protoplasts using the PEG‐mediated method as previously described (Zheng *et al.*, 2014). After a 16‐h incubation, GFP fluorescence was monitored using a LSM880 laser‐scanning confocal microscope (Carl Zeiss, Jena, Germany).

### Generation of overexpression and RNAi lines

For the *TaDA1* overexpression construct, the full‐length *TaDA1‐A* CDS was amplified from Chinese Spring seedling cDNA sample and cloned into the *Bam*HI and *Spe*I sites of the pWMB006 vector under the control of the maize ubiquitin promoter *Ubi* (Wang *et al.*, 2017b). The resulting construct was double‐digested using *Hind*III and *Eco*RI, and then cloned into the corresponding sites of the pCAMBIA3301 vector to generate the *TaDA1* overexpression construct. To generate the *TaDA1* RNAi construct, a 403‐bp sequence derived from the *TaDA1‐A* CDS (nucleotides 418–820) was amplified and sequentially cloned into the *Bam*HI/*Kpn*I and *Sac*I/*Spe*I sites of the pWMB006 vector. The resulting construct was double‐digested using *Hind*III and *Eco*RI, and then cloned into the corresponding sites of the pCAMBIA3301 vector to generate the *TaDA1* RNAi construct. Similarly, a 402‐bp sequence from the *TaGW2‐A* CDS (nucleotides 302–703) was used to generate the *TaGW2* RNAi construct.

All the constructs were introduced into *Agrobacterium tumefaciens* strain EHA105 and transformed into immature embryos of wheat (variety Fielder) following a modified *Agrobacterium*‐mediated transformation method (Wang *et al.*, 2017b). The phenotypic traits of the T_2_ generation transgenic lines were measured at the Chinese Academy of Agricultural Sciences experimental field in Beijing, China. To assess phenotypic differences between transgenic lines and wild type, we performed a one‐way analysis of variance (ANOVA) using SPSS 16.0 (IBM Corporation, Armonk, NY) and a Tukey test at a significance level of *P* < 0.05.

### Microscopy and measurement of cell length

The 15‐DPA developing grains were collected from *DA1‐OE‐1*, *DA1‐Ri‐3*, and WT plants, fixed for 24 h in FAA solution (70% alcohol, 5% acetic acid, and 0.02% formaldehyde; v/v), dehydrated in a series of ethanol solutions (75%, 85%, 90%, 95%, and 100%; v/v), and infiltrated with Paraplast Regular (Sigma‐Aldrich, St. Louis, MO). Tissue sections (4‐μm thick) were cut on a microtome (Leica Microsystems, Nussloch, Germany), stained using periodic acid–Schiff reagent for 30 min, and then counter‐stained with Luxol Fast Blue stain, as described previously (Wu *et al.*, 2016). The sections were imaged under a SteREO Discovery V20 microscope (Carl Zeiss). To measure the cell length, approximately 100 outer pericarp cells were analysed in each of at least six different grain sections using the ZEN 2012 Image Processing software (Carl Zeiss).

### TaDA1‐A haplotype discovery and association analysis

A total of 36 wheat accessions (24 modern cultivars and 12 landraces) were initially analysed for any *TaDA1‐A* sequence variations (Table S3). Specific primers were designed to amplify the 2.1‐kb promoter region and the 6.4‐kb coding region of *TaDA1‐A* (Table S2). The resulting DNA fragments were purified and sequenced on an Applied Biosystems 3730XI DNA Analyzer (Thermo Fisher Scientific, Carlsbad, CA). Sequence variations (SNPs and InDels) were identified using DNASTAR (http://www.dnastar.com/). Molecular markers of the *TaDA1‐A* haplotypes were developed based on two SNPs (T/A at 2590 bp and G/A at 4437 bp). A previously described method (Hou *et al.*, 2014; Ma *et al.*, 2016) was used to perform the haplotype association analysis. Briefly, the variance analyses were performed using SPSS 16.0 (IBM Corporation). Phenotypic differences between haplotypes were determined using a one‐way ANOVA and a Tukey test at a significance level of *P* < 0.05.

### Transient promoter activity assay

The transient promoter activity assay was performed using a dual‐luciferase system in *N. benthamiana* leaves as described (Hellens *et al.*, 2005). Briefly, the 2.1‐kb promoter sequences from different *TaDA1‐A* haplotypes were cloned into the pGreenII 0800‐Luc reporter plasmid. The resulting derivative constructs and pSoup‐p19 were transformed into *Agrobacterium tumefaciens* strain GV3101, and then co‐infiltrated into *N. benthamiana* leaves. After 48 h incubation, the assay was performed using the Dual‐Luciferase Reporter Assay System (Promega, Madison, WI), and the relative LUC activity (LUC/REN) was measured by a GloMax‐Multi luminescence reader (Promega).

### Yeast two‐hybrid assay and LCI assay

The yeast two‐hybrid assay was carried out as described previously (Wu and Li, 2017). The *TaDA1‐A* and *TaGW2‐B* CDSs were amplified and sub‐cloned into the pGBKT7 and pGADT7 vectors, respectively, to generate the *TaDA1‐A‐BD* and *TaGW2‐B‐AD* plasmids. After the co‐transformation of both plasmids into yeast AH109 cells, the interaction between the expressed proteins was determined by the growth of the co‐transformants on a selection medium (SD/‐Trp/‐Leu/‐His/‐Ade), following the Yeast Protocols Handbook (Takara Bio).

The LCI assay for the interaction between TaDA1‐A and TaGW2‐B was performed in *N. benthamiana* leaves as described previously (Liu *et al.*, 2017). The full‐length *TaDA1‐A* and *TaGW2‐B* CDSs were fused with the *N*‐terminal and *C*‐terminal regions of the *LUC* reporter gene, respectively (*TaDA1‐A‐nLUC* and *cLUC‐TaGW2‐B*), and transformed into *Agrobacterium tumefaciens* strain GV3101. *Agrobacteria* harbouring the nLUC and cLUC derivative constructs were co‐infiltrated into *N. benthamiana*, and the LUC activity was imaged and analysed 48–72 h after infiltration using the NightSHADE LB 985 Plant Imaging System (Berthold Technologies, Bad Wildbad, Germany).

### Protein expression and in vitro pull‐down assay

The full‐length *TaDA1‐A* and *TaGW2‐B* CDSs were amplified and inserted into the pMAL‐c2X (New England Biolabs, Beverly, MA) and pET32a (Merck Millipore, Darmstadt, Germany) vectors, respectively. The resulting plasmids were transformed into *Escherichia coli* strain Rosetta (DE3) and induced using 0.4 mm isopropyl β‐D‐thiogalactopyranoside (IPTG) for 6 h at 20 °C. For the protein purification, the cultured cells were centrifuged at 6000 g at 4 °C for 5 min and then lysed using sonication. MBP‐TaDA1‐A and MBP proteins were purified using amylose resin (New England Biolabs) and the His‐TaGW2‐B protein was purified using Ni‐NTA resin (Qiagen, Hilden, Germany), according to the manufacturer’s instructions.

The *in vitro* pull‐down assay was performed as described previously (Dong *et al.*, 2018). Briefly, 2 μg His‐TaGW2‐B protein was mixed with 2 μg MBP‐TaDA1‐A or MBP protein in 500 μl binding buffer [20 mm Tris (pH 7.4), 200 mm NaCl, 1 mm EDTA, 1 mm PMSF, 1 mm DTT, and 1 × protease inhibitor (Roche, Indianapolis, IN)], and then pulled down overnight using amylose resin (New England Biolabs) at 4 °C. The amylose resin was washed six times with binding buffer, and the proteins that bound to the resin were eluted by boiling the samples in 50 μl SDS‐PAGE loading buffer for 5 min. The eluted proteins were separated using a 12% SDS‐PAGE and detected by immunoblotting with anti‐MBP and anti‐His antibodies (CWbiotech, Beijing, China).

### Proteome analysis

Approximately 2 g of developing 10‐DPA grains were collected from *DA1‐Ri‐3*, *GW2‐Ri‐3*, and WT plants and used for protein extraction. The total proteins were extracted from three biological replicates using extraction buffer (8 m urea, 30 mm HEPES, 1 mm PMSF, 2 mm EDTA, 10 mm DTT, and 55 mm IAM). Protein digestion was performed following the filter‐aided sample preparation (FASP) method, as described previously (Wiśniewski *et al.*, 2009). Peptides from each sample were labelled with isobaric mass tags for relative and absolute quantitation (iTRAQ) using an iTRAQ Reagent‐8Plex Multiplex Kit (Sciex, Framingham, MA).

The liquid chromatography–mass spectrometry (LC‐MS)/MS analysis was performed on a Q Exactive mass spectrometer (Thermo Fisher Scientific) coupled with a Dionex Ultimate 3000 Nano LC system (Thermo Fisher Scientific). The mass spectrometer was operated in a data‐dependent mode with a full MS scan from 350–2000 m/z, a full scan resolution at 70 000, and MS/MS scan resolution at 17 500. The MS/MS spectra were searched against the IWGSC *Triticum aestivum* protein database (http://plants.ensembl.org/index.html) using the SEQUEST engine and quantified using Proteome Discoverer (version 1.4; Thermo Fischer Scientific). Differently abundant proteins between the WT and *DA1‐Ri‐3* or *GW2‐Ri‐3* (in each of the three biological replicates) were defined as having a ratio of ≥1.2 or ≤0.8 between samples, determined using an ANOVA at a significance level of *P* < 0.05.

## Conflict of interest

The authors declare that they have no conflict of interests.

## Author contributions

X.Z, T.L., and C.H. designed the research and analysed the data; H.L., T.L., H.F.L., K.W., Y.W., and L.Q. performed the experiments; T.L. and H.L. wrote the article; X.Z. supervised and revised the writing of the article; D.A. contributed during revision, providing an F_16_ segregating population and phenotype data used for association analysis.

## Supporting information


**Figure S1** PCR‐based chromosome mapping of the three *TaDA1* homoeologs in Chinese Spring (CS) nullisomic‐tetrasomic lines.
**Figure S2** Gene structures of the *TaDA1* homoeologs.
**Figure S3** Sequence alignment of DA1 proteins from wheat, *Arabidopsis*, and maize.
**Figure S4** Comparison of other yield‐related traits between the WT and *TaDA1* transgenic lines under field conditions.
**Figure S5** Comparison of cell number and cell length in the outer pericarp tissues of *DA1‐OE‐1*, WT, and *DA1‐Ri‐3*.
**Figure S6** Development of molecular markers for the *TaDA1‐A* haplotypes based on the T/A SNP at position 2590 and the G/A SNP at position 4437.
**Figure S7** The effect of *TaDA1‐A* haplotypes on TKW in an F_16_ segregation population derived from Chuan 35050 × Shannong 483.
**Figure S8** Biochemical functional analysis of proteins encoded by the three *TaDA1‐A* haplotypes.
**Figure S9** Global selection and frequency change of the *TaDA1‐A* haplotypes in modern cultivars.
**Figure S10** Sequence alignment of GW2 proteins from wheat and rice, and the DA2 protein from *Arabidopsis*.
**Figure S11** The down‐regulation of *TaGW2* led to significantly increased kernel sizes and weights in wheat.

**Table S1** Gene IDs or accession numbers

**Table S2** Primers used in this study

**Table S3** The 36 wheat accessions used for polymorphism discovery

**Table S4** Genotype and phenotype of the 348 Chinese modern cultivars

**Table S5** Association results of the phenotypes and *TaDA1‐A* haplotypes of 348 modern cultivars grown in three different environments

**Table S6** Differently abundant proteins in the 10‐DPA grains of *DA1‐Ri‐3*, *GW2‐Ri‐3*, and the WT
