# Supplementary material for: TaDA1, a conserved negative regulator of kernel size, has an additive effect with TaGW2 in common wheat (Triticum aestivum L.)
Source: Plant Biotechnol J. 2019 Dec 4;18(5):1330–42. doi: 10.1111/pbi.13298 (PMC7152612; doi:10.1111/pbi.13298)
Supplement: Supplementary file 1 — Figure S1 PCR‐based chromosome mapping of the three TaDA1 homoeologs in Chinese Spring (CS) nullisomic‐tetrasomic lines. Figure S2 Gene structures of the TaDA1 homoeologs. Figure S3 Sequence alignment of DA1 proteins from wheat, Arabidopsis, and maize. Figure S4 Comparison of other yield‐related traits between the WT and TaDA1 transgenic lines under field conditions. Figure S5 Comparison of cell number and cell length in the outer pericarp tissues of DA1‐OE‐1, WT, and DA1‐Ri‐3. Figure S6 Development of molecular markers for the TaDA1‐A haplotypes based on the T/A SNP at position 2590 and the G/A SNP at position 4437. Figure S7 The effect of TaDA1‐A haplotypes on TKW in an F16 segregation population derived from Chuan 35050 × Shannong 483. Figure S8 Biochemical functional analysis of proteins encoded by the three TaDA1‐A haplotypes. Figure S9 Global selection and frequency change of the TaDA1‐A haplotypes in modern cultivars. Figure S10 Sequence alignment of GW2 proteins from wheat and rice, and the DA2 protein from Arabidopsis. Figure S11 The down‐regulation of TaGW2 led to significantly increased kernel sizes and weights in wheat. [file PBI-18-1330-s006.pdf]

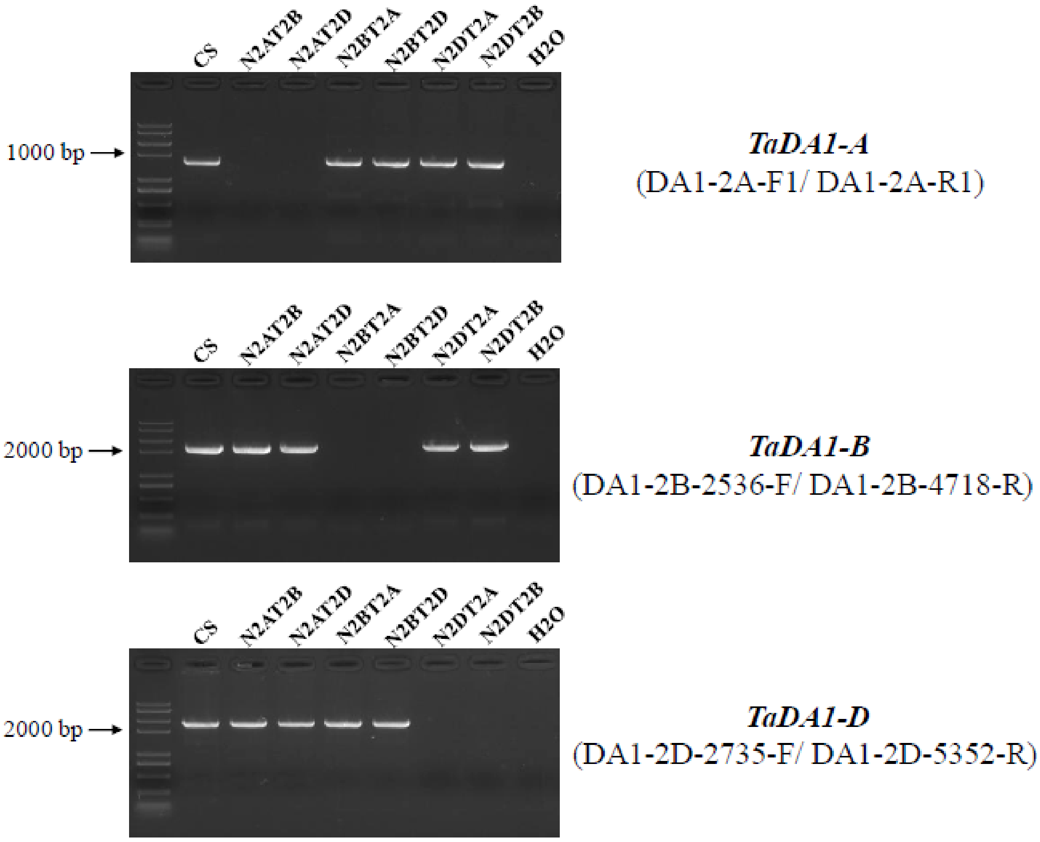

Figure S1. PCR-based chromosome mapping of the three *TaDA1* homoeologs in Chinese Spring (CS) nullisomic-tetrasomic lines. Primers used are shown in the brackets.

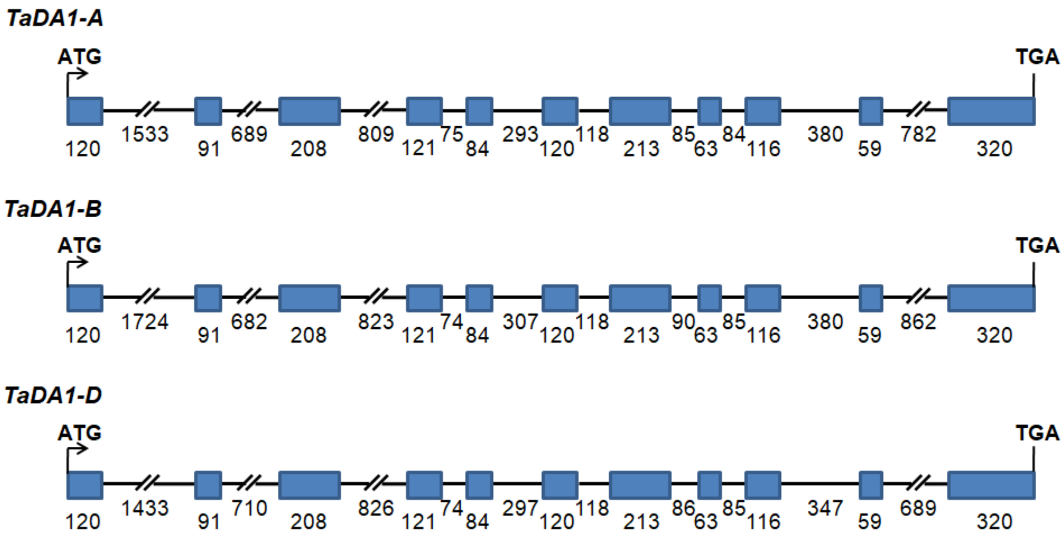

Figure S2. Gene structures of the *TaDA1* homoeologs. The blue boxes indicate exons.

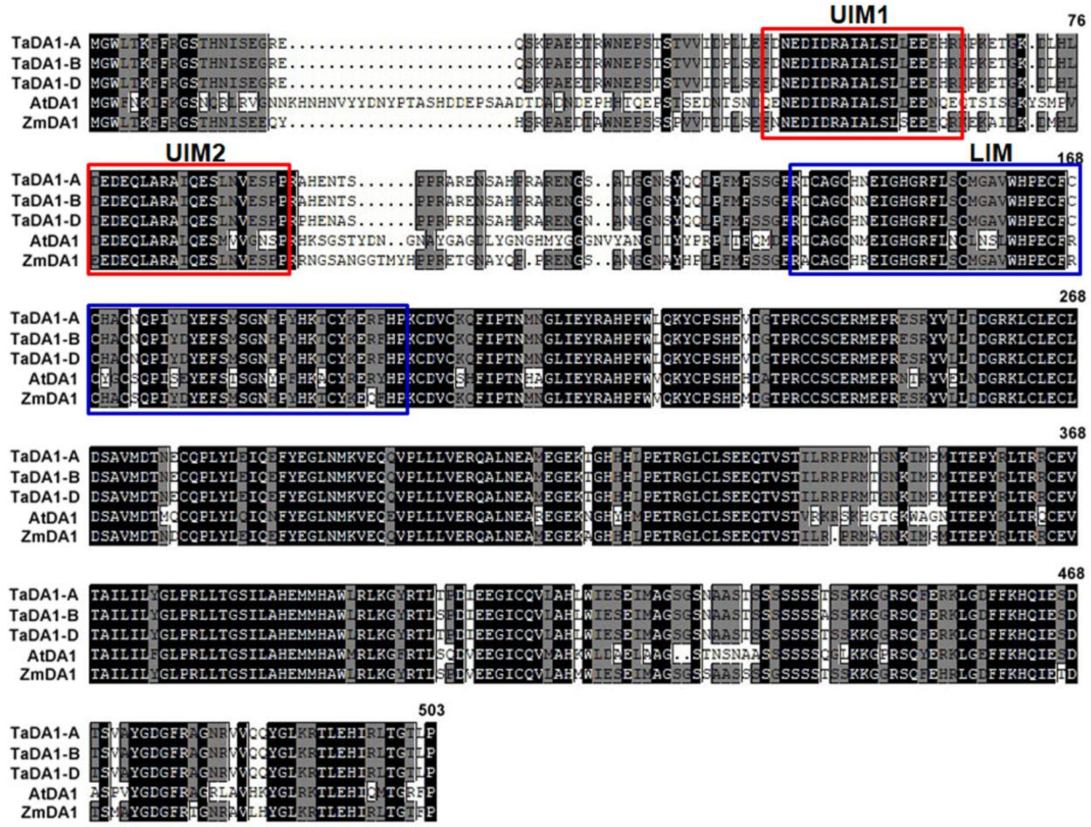

Figure S3. Sequence alignment of DA1 proteins from wheat, *Arabidopsis*, and maize. UIM, ubiquitin interaction motif; LIM, Lin11/Isl-1/Mec-3.

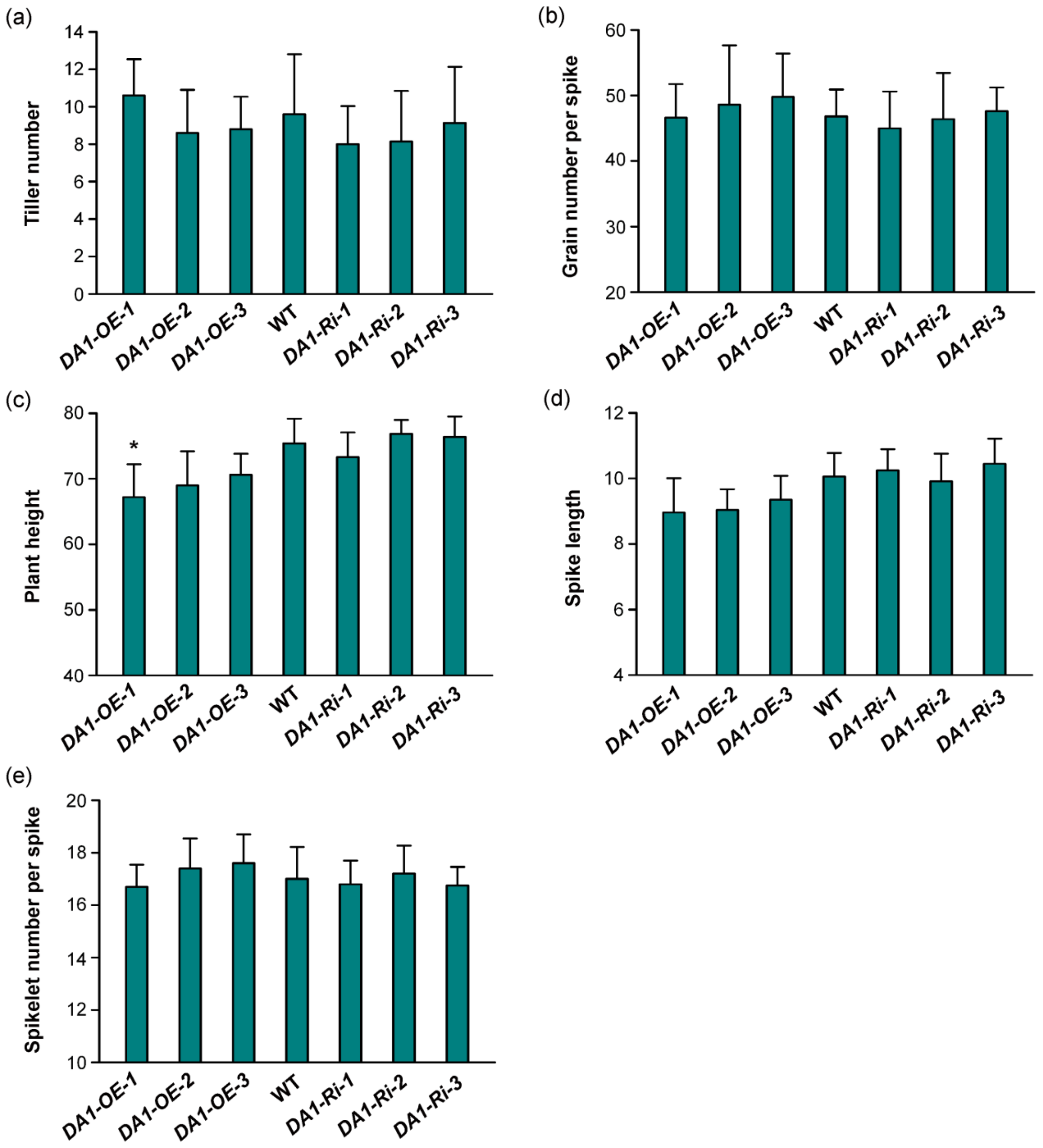

Figure S4. Comparison of other yield-related traits between the WT and *TaDA1* transgenic lines under field conditions. (a) Tiller number; (b) Grain number per spike; (c) Plant height; (d) Spike length; (e) Spikelet number per spike. $^{*}P < 0.05$ (ANOVA) indicates a significant difference with the WT.

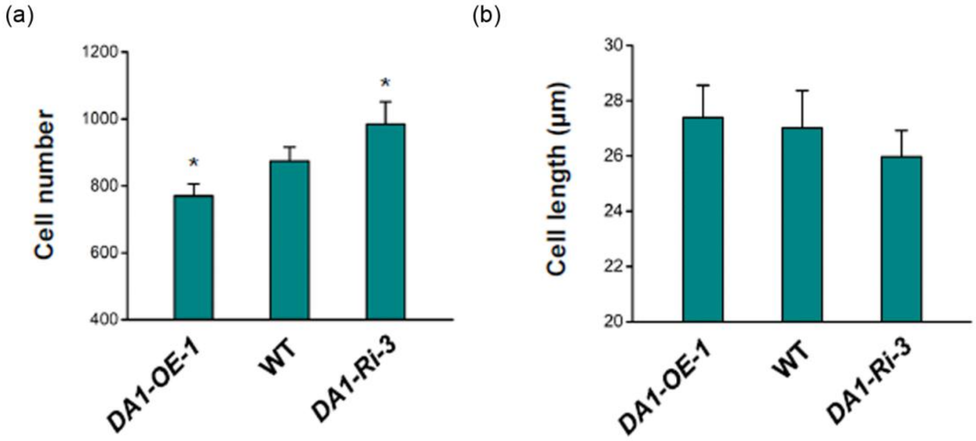

Figure S5. Comparison of cell number (a) and cell length (b) in the outer pericarp tissues of *DA1-OE-1*, WT, and *DA1-Ri-3*. The values are presented as mean ± SD. $^{*}P < 0.05$ (ANOVA) indicates a significant difference with the WT.

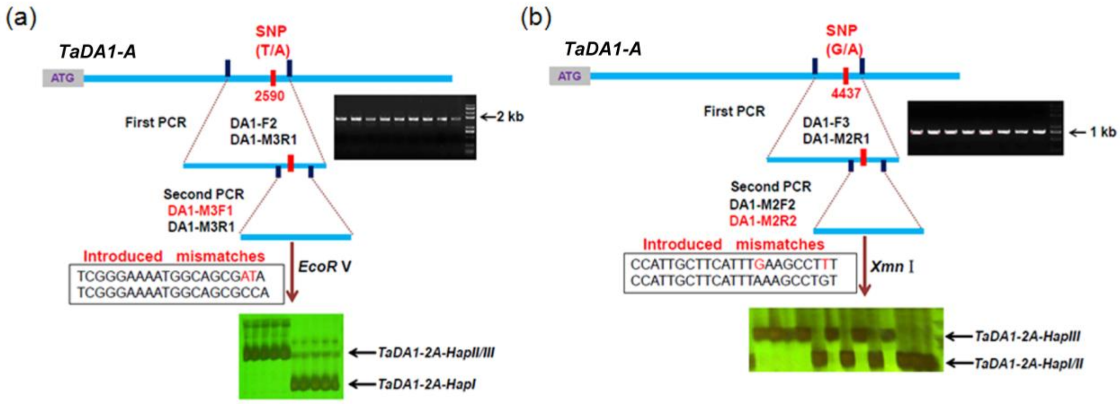

Figure S6. Development of molecular markers for the *TaDA1-A* haplotypes based on the T/A SNP at position 2,590 (a) and the G/A SNP at position 4,437 (b).

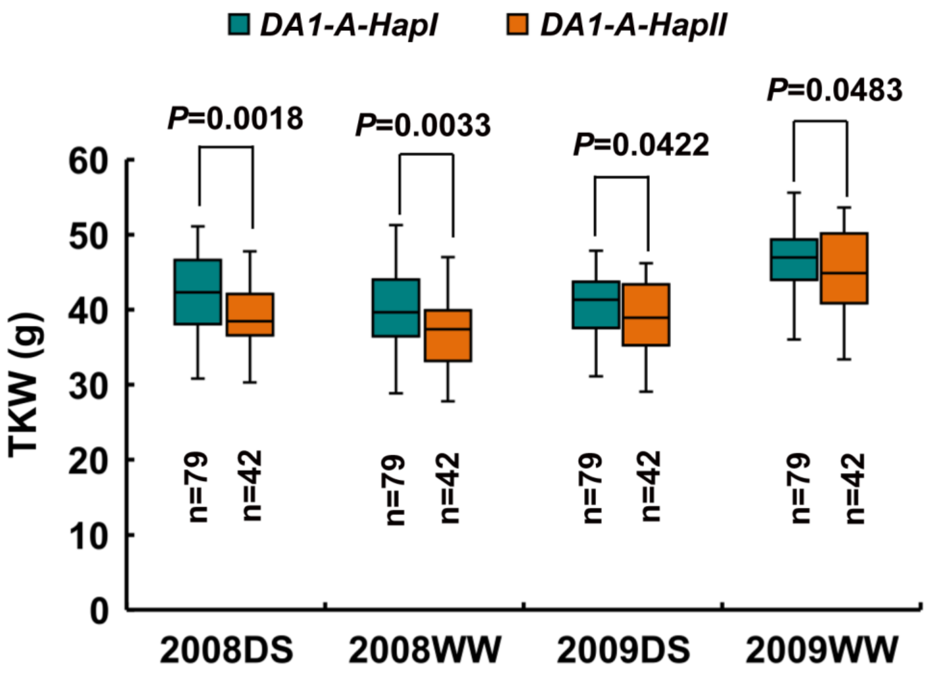

Figure S7. The effect of *TaDA1-A* haplotypes on TKW in an F_{16} segregation population derived from Chuan 35050 × Shannong 483. Association of *TaDA1-A* haplotypes with TKWs of the segregation populations in four environments including well watered control (2008WW and 2009WW) and drought stress (2008DS and 2009DS). The number of *TaDA1-A-HapI* and *TaDA1-A-HapII* lines is 79 and 42, respectively. Box plots showing the components of descriptive statistics including sample size (n), medians (black lines), upper and lower quartiles (box edges), minimums and maximums (whiskers), and *P* values (ANOVA).

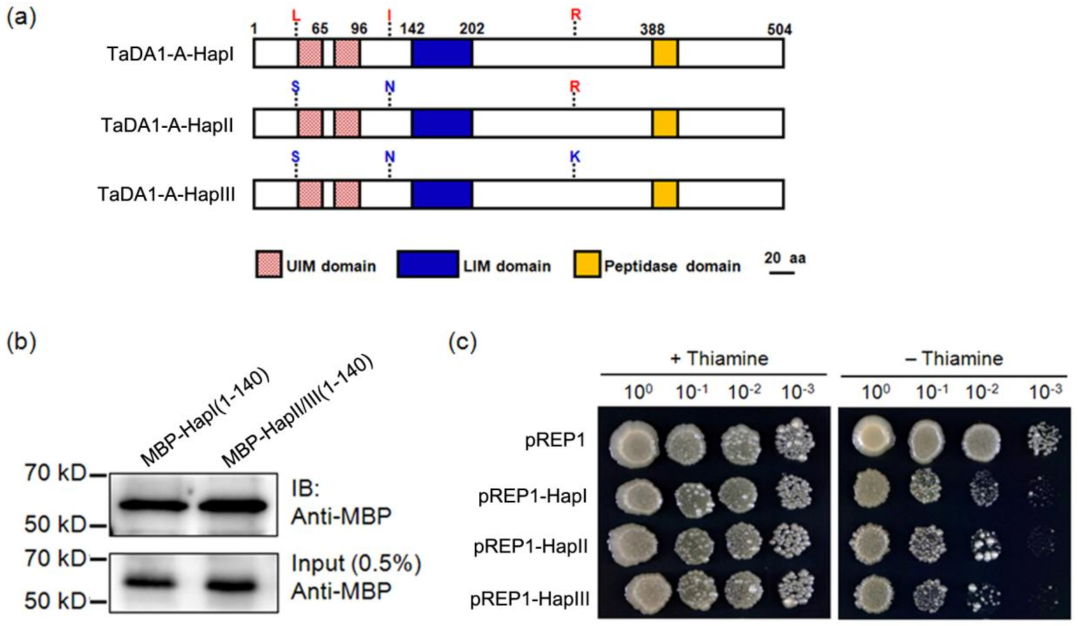

Figure S8. Biochemical functional analysis of proteins encoded by the three *TaDA1-A* haplotypes. (a) Three amino acids mutations (L44S, I123N, R307K) present among different TaDA1-A haplotypes. The conserved domains were shown in different boxes. (b) *In vitro* ubiquitin pull down assay showing that both HapI and HapII/III proteins had similar activity in binding ubiquitin. MBP fused N-terminal TaDA1-A-HapI and -HapII/III proteins (amino acids 1-140) containing UIM domains were expressed in *Escherichia coli* and incubated with ubiquitin-agarose beads. The beads were washed, and proteins remaining on the beads were resolved by SDS-PAGE and identified by Western blotting using MBP antibody as decribed by Li et al. (2008). (c) Growth of *S. pombe* transformed with the empty vector pREP1 and pREP1-TaDA1-A-HapI/II/III in the presence (+ thiamine, the expression of HapI/II/III is repressed in the presence of thiamine) or absence of thiamine (– thiamine, the expression of HapI/II/III is induced in the absence of thiamine) as described by Lin *et al.* (2012).

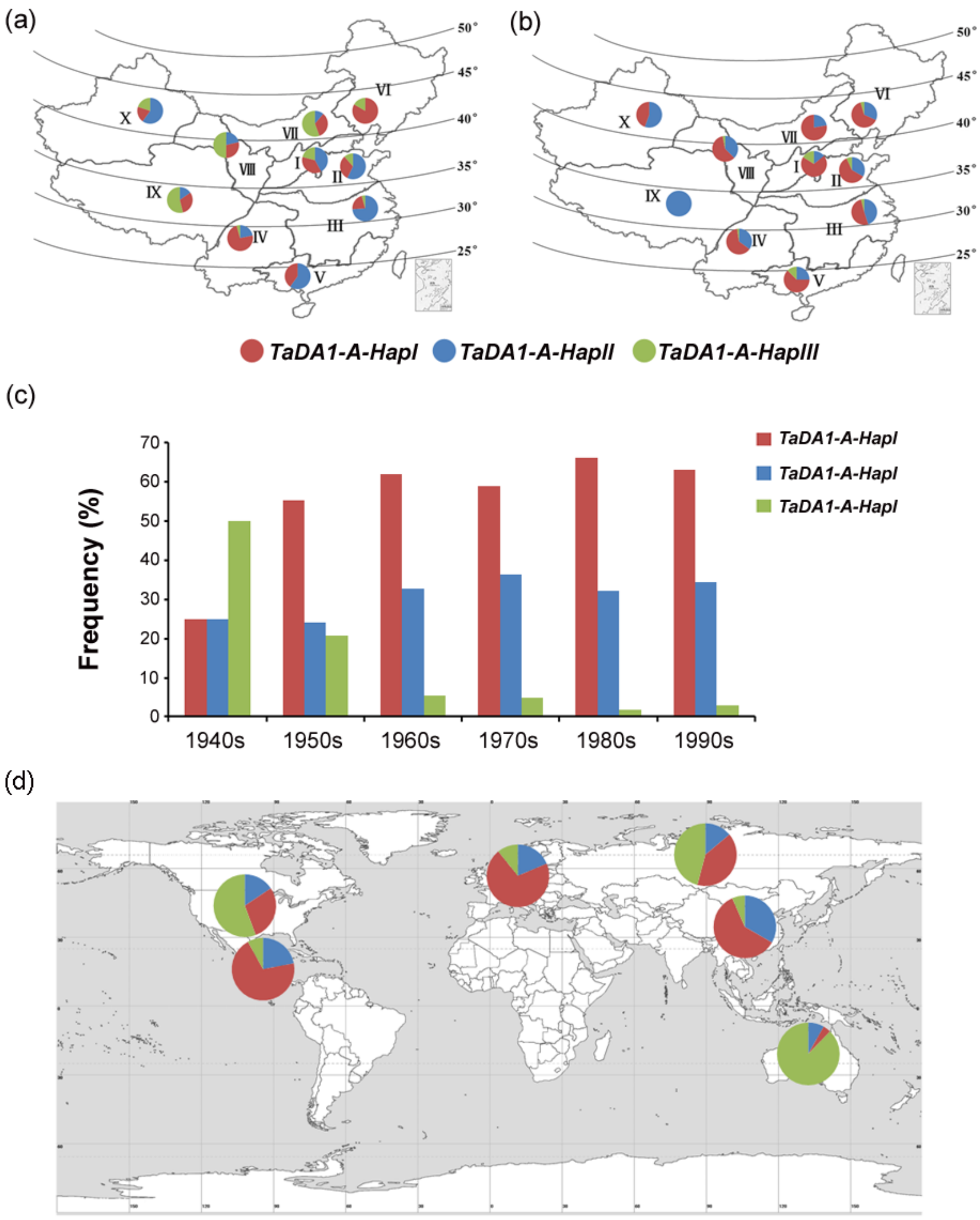

Figure S9. Global selection and frequency change of the *TaDA1-A* haplotypes in modern cultivars. (a, b) Geographic distribution of *TaDA1-A* haplotypes in Chinese landraces (a) and modern cultivars (b). I–X indicate China's ten ecological zones. (c) Frequency change of the *TaDA1-A* haplotypes in the Chinese cultivars developed between the 1940s and the 1990s. (d) Global distribution of *TaDA1-A* haplotypes in modern cultivars from China, Europe, the former USSR, America, CIMMYT, and Australia.

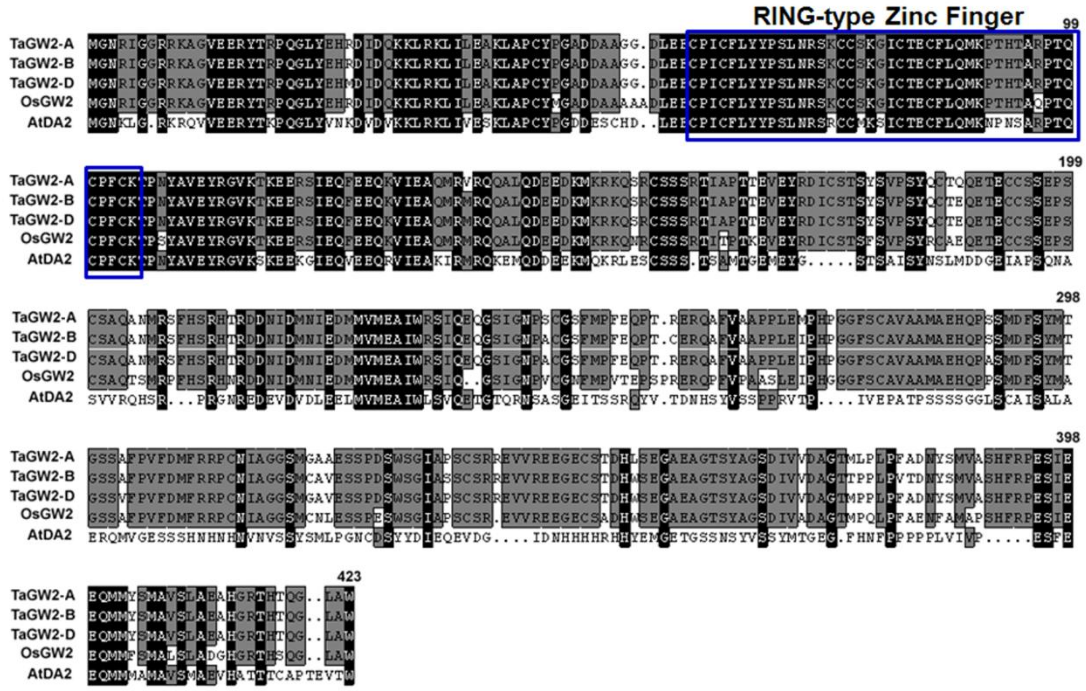

Figure S10. Sequence alignment of GW2 proteins from wheat and rice, and the DA2 protein from *Arabidopsis*.

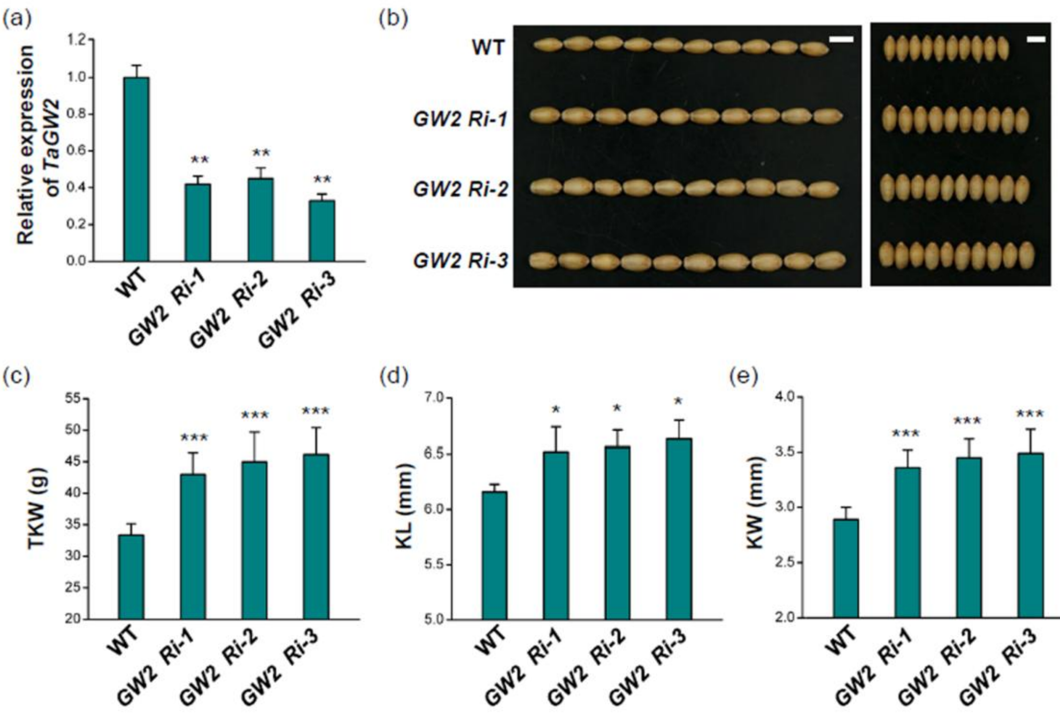

Figure S11. The downregulation of *TaGW2* led to significantly increased kernel sizes and weights in wheat. (a) Expression levels of *TaGW2* in WT and three independent *TaGW2* RNAi lines (*GW2 Ri1–Ri3*). (b) Comparison of kernel phenotype between WT and *TaGW2* RNAi lines. Bar = 5 mm. (c) TKW, (d) KL, and (e) KW of WT and *TaGW2* RNAi lines. $^{*}P < 0.05$, $^{**}P < 0.01$ and $^{***}P < 0.001$ (ANOVA).
