# Supplementary material for: TaDA1, a conserved negative regulator of kernel size, has an additive effect with TaGW2 in common wheat (Triticum aestivum L.)
Source: Plant Biotechnol J. 2019 Dec 4;18(5):1330–42. doi: 10.1111/pbi.13298 (PMC7152612; doi:10.1111/pbi.13298)
Supplement: Supplementary file 3 — Table S2 Primers used in this study [file PBI-18-1330-s004.docx]

**Table S2.** Primers used in this study.

| **Primers used for polymorphism discovery and marker development (5′-3′)** | |
| --- | --- |
| DA1-2A-F1 | ACCTTCCTTTAGTTCTGCATTG |
| DA1-2A-R1 | TACTACCTCCGTCCAGAATAAACT |
| DA1-2A-F2 | TCCGGAATTAATTGTTGCTGAA |
| DA1-2A-R2 | TCCAGGCAGAGTTTACGACCA |
| DA1-2A-F3 | ACATAAGTTGCTGTCTCCAAACC |
| DA1-2A-R3 | CTGCAATCTTAGCACGCAGTAT |
| DA1-2A-pF | TCGCGTTTCAACATTGTTTATGG |
| DA1-2A-pR | AAGGATGGCATACAATCATCAAGC |
| DA1-M2R1 | ATCAGTAACTGTTTCTATTGC |
| DA1-M2F2 | ATGTATTGCTTGATGATGGTCGT |
| DA1-M2R2 | CCATTGCTTCATTTGAAGCCTTT |
| DA1-M3F1 | TCGGGAAAATGGCAGCGATA |
| DA1-M3R1 | AGCAGTTCAAATGTGTGGCACT |
| DA1-2B-2536-F | GCTCATTGTATTCTCCAGTCTTCC |
| DA1-2B-4718-R | TAATGGTGGTTCAGTTCAGCATAAC |
| DA1-2D-2735-F | CTTCTGATAAGTTGGATTGGCTCTG |
| DA1-2D-5352-R | AGTATTACAAACTAAATGCATGGAT |
| **Primers used for qRT-PCR (5**′**-3**′**)** | |
| DA1-ABD-qRT-F  DA1-ABD-qRT-R  DA1-A-qRT-F | GATACAAATGAGTGCCAGCCT  ACGGCTCTGTTATCATTTCCA  CGACTTAAAGGATACCGCACG |
| DA1-A-qRT-R | GGATGTGGATGACGAAGAGGAC |
| DA1-B-qRT-F | CGACTTAAAGGATACCGCACT |
| DA1-B-qRT-R | CTTCTTCGACGATGCGGAC |
| DA1-D-qRT-F | CGTCTTAAAGGATACCGCACA |
| DA1-D-qRT-R | TTCTTCGACGATGTGGATGAT |
| GW2-RT-F | TGGTTATGGAAGCGATTTGG |
| GW2-RT-R | CCATAGCAGCAACAGCACAA |
| TaActin-F | AGGTGCCCTGAGGTGCTGTT |
| TaActin-R | GCCAAAATAGAGCCACCGAT |
| **Primers for plasmid constructing (5′-3′)** | |
| DA1-OE-F (for DA1 OE) | CGGGATCCATGGGTTGGTTGACCAAGTT |
| DA1-OE-R (for DA1 OE) | ACGCACTAGTTCAGAATGGCAATGTCCCTG |
| DAi-BamHI-F (for DA1 RNAi) | CGGGATCCAGGACATGTGCTGGATGTCA |
| DAi-KpnI-R (for DA1 RNAi) | GGGGTACCCCATCACTGCAGAGTCAAGG |
| DAi-SacI-F (for DA1 RNAi) | ACGCGAGCTCAGGACATGTGCTGGATGTCA |
| DAi-SpeI-R (for DA1 RNAi) | ACGCACTAGTCCATCACTGCAGAGTCAAGG |
| GWi-BamHI-F (for GW2 RNAi) | CGGGATCCCATTCTGCAAAACCCCCA |
| GWi-KpnI-R (for GW2 RNAi) | GGGGTACCAACGCCAAATCGCTTCCA |
| GWi-SacI-F (for GW2 RNAi) | ACGCGAGCTCCATTCTGCAAAACCCCCA |
| GWi-SpeI-R (for GW2 RNAi) | ACGCACTAGTAACGCCAAATCGCTTCCA |
| DA1-CLUC-F | GGGGTACCATGGGTTGGTTGACCAAG |
| DA1-CLUC-R | ACGCGTCGACTCAGAATGGCAATGTCCC |
| GW2-NLUC-F | GGGGTACCATGGGGAACAGAATAGGA |
| GW2-NLUC-R | ACGCGTCGACCAACCATGCCAACCCTTG |
| DA1-BD-F | CGGAATTCGAAAATACCTCACCCCCT |
| DA1-BD-R | CGGGATCCTCAGAATGGCAATGTCCCTG |
| GW2-AD-F | ACGCCATATGATGGGGAACAGAATAGGAGG |
| GW2-AD-R | CGGGATCCTTACAACCATGCCAACCCT |
