# Supplementary material for: TaDA1, a conserved negative regulator of kernel size, has an additive effect with TaGW2 in common wheat (Triticum aestivum L.)
Source: Plant Biotechnol J. 2019 Dec 4;18(5):1330–42. doi: 10.1111/pbi.13298 (PMC7152612; doi:10.1111/pbi.13298)
Supplement: Supplementary file 6 — Table S5 Association results of the phenotypes and TaDA1‐A haplotypes of 348 modern cultivars grown in three different environments [file PBI-18-1330-s003.docx]

**Table S5.** Association results of the phenotypes and *TaDA1-A* haplotypes of 348 modern cultivars grown in three different environments.

|  | **2002LY** | | | **2005LY** | | | **2010SY** | | |
| --- | --- | --- | --- | --- | --- | --- | --- | --- | --- |
|  | ***HapI*** | ***HapII*** | ***HapIII*** | ***HapI*** | ***HapII*** | ***HapIII*** | ***HapI*** | ***HapII*** | ***HapIII*** |
| **HD (d)** | 176.30±0.41a | 177.07±0.58a | 178.68±1.14a | 198.96±0.24a | 200.11±0.43b | 200.14±0.57ab | 217.15±0.22A | 218.42±0.35B | 217.57±0.61AB |
| **MD (d)** | 225.66±0.59a | 227.59±0.78a | 228.05±1.40a | 236.83±0.24a | 237.76±0.38a | 237.36±0.82a | 252.83±0.20A | 253.92±0.31B | 254.17±0.69AB |
| **SL (cm)** | 10.68±0.17a | 10.42±0.21a | 9.76±0.36a | 9.28±0.11a | 9.26±0.19a | 8.28±0.31b | 10.06±0.11a | 9.92±0.16ab | 9.14±0.27b |
| **SN** | 21.34±0.18a | 21.09±0.29a | 21.00±0.53a | 21.30±0.14a | 21.42±0.21a | 20.39±0.29a | 20.77±0.14a | 20.66±0.20a | 19.96±0.25a |
| **PH (cm)** | 91.70±1.48a | 91.73±2.11a | 102.82±4.07a | 93.41±1.22A | 89.90±1.84A | 106.82±2.94B | 91.00±1.14a(AB) | 87.88±1.71a(A) | 101.72±2.81b(B) |
| **GN** | 52.46±0.85ab | 53.58±1.05a | 46.63±2.38b | 47.48±0.60A | 47.87±0.91A | 41.11±1.34B | 54.05±0.63a | 54.05±0.91a | 48.16±1.50b |
| **ETN** | 7.14±0.20a | 7.17±0.27a | 8.21±0.69a | 8.83±0.25a | 9.09±0.35a | 10.53±0.76a | 11.42±0.26a | 12.12±0.37a | 12.79±0.74a |
| **TKW (g)** | 43.78±0.49a(A) | 40.67±0.68b(B) | 39.43±1.32b(AB) | 40.50±0.46a | 38.59±0.65b | 36.25±1.30b | 40.70±0.46a | 38.75±0.54b | 38.50±1.20ab |
| **KL (mm)** | 6.77±0.03a | 6.74±0.05a | 6.53±0.10a | 6.93±0.03a | 6.78±0.05b | 6.62±0.07b | 6.93±0.03a | 6.81±0.04a | 6.71±0.07a |
| **KW (mm)** | 3.37±0.02a | 3.37±0.02a | 3.22±0.05b | 3.31±0.01a | 3.26±0.02ab | 3.19±0.05b | 3.24±0.02a | 3.22±0.02a | 3.14±0.04a |
| **KT (mm)** | 2.89±0.02a | 2.87±0.02a | 2.78±0.05a | 2.93±0.04a | 2.89±0.02a | 2.87±0.04a | 3.06±0.06a | 2.98±0.01a | 3.21±0.25a |

Different capital and small letters indicate significant differences between haplotypes at *P* < 0.01 and *P* < 0.05, respectively. N_HapI_ = 209; N_HapII_ = 116; N_HapIII_ = 23.

HD, heading date; MD, maturity date; SL, spike length; SN, spikelet number per spike; PH, plant height; GN, grain number per spike; ETN, effective tiller number; TKW, thousand-kernel weight; KL, kernel length; KW, kernel width; KT, kernel thickness.
